# Systematic review: the impact of maternal pre-and postnatal cannabis use on the behavioral and emotional regulation in early childhood

**DOI:** 10.1007/s00787-024-02494-8

**Published:** 2024-06-15

**Authors:** Emely Reyentanz, Jennifer Gerlach, Sören Kuitunen-Paul, Yulia Golub

**Affiliations:** 1 Department of Child and Adolescent Psychiatry, Carl Von Ossietzky Universität Oldenburg, Oldenburg, Germany; 2 Department of Child and Adolescent Mental Health, University Hospital Erlangen, Friedrich-Alexander-Universität Erlangen-Nürnberg, Erlangen, Germany; 3 Chair of Clinical Child and Adolescent Psychology and Psychotherapy, Technische Universität Chemnitz, Chemnitz, Germany; 4 Chair of Clinical Psychology and Psychotherapy, Technische Universität Chemnitz, Chemnitz, Germany; 5 Chair of Child and Adolescent Psychiatry and Psychotherapy, Technische Universität Dresden, Dresden, Germany

**Keywords:** Cannabis exposure, Self-regulation, Neurobiological mechanisms, Early childhood, Systematic review

## Abstract

Prenatal exposure to alcohol and tobacco has been associated with child regulatory abilities and problems, but less is known about the associations with cannabis exposure. This review seeks to address this gap primarily focusing on the effects of maternal cannabis use on the child. Thus, we investigate the association between pre- and postnatal cannabis exposure of the child and regulatory abilities and problems, as well as the underlying neurobiological mechanisms potentially mediating the associations. According to the PRISMA guidelines, a systematic literature review was performed based on a systematic literature search through Medline (PubMed), Web of Science and PsycInfo, including studies assessing children aged 0–6 years with cannabis exposure in the preconception, pre-or postnatal period (preconception, pre- and postnatal cannabis exposure [PCE]) and investigating child regulatory abilities, regulatory problems or neurobiological mechanisms. Of *n* = 1061 screened articles, *n* = 33 were finally included. Diminished regulatory abilities are more likely to be found in infants after PCE, while specific regulatory problems tend to be more frequently found after two years of age. Possible mechanisms are related to changes in methylation and expression of key genes involved in endocannabinoid, dopaminergic and opioid systems, increased cortisol reactivity and altered Secretory Immunoglobulin A levels. Furthermore, PCE has been associated with changes in brain structure and connectivity. Current findings indicate that PCE is associated with both age-dependent alterations in self-regulation and neurobiological changes in young children. However, evidence is limited due to the number of studies, small sample sizes and lack of control for maternal psychopathology. Longitudinal studies including psychometric data from mothers are needed in order to further understand the implications of PCE.

*Trial registration***:** The review is registered with PROSPERO (ID: CRD42023425115).

**Supplementary Information:**

The online version contains supplementary material available at 10.1007/s00787-024-02494-8.

## Introduction

After alcohol and tobacco, cannabis is the most commonly used drug [1, 2] and its use has increased in recent years [3], including during pregnancy [4]. The prevalence of cannabis use during pregnancy varies between studies depending on sample characteristics and methods used, and ranges from 2% to almost 30% [5, 6], with the highest prevalence in the first trimester [5]. In recent years, a growing number of countries have legalized the recreational use of cannabis [7]. A recent review indicates that cannabis use increases in countries where cannabis is legalized for recreational use, not only among adults in general, but also among pregnant women [8]. Numerous studies have demonstrated the negative effects of prenatal substance exposure, e.g. alcohol (PAE) [9, 10] and tobacco (PTE) [11, 12], on the psychological development of children whereas the effects of cannabis exposure are less well investigated. Previous studies indicate a higher risk for externalizing problems [13, 14], aggressive behavior, attention deficit hyperactivity disorder (ADHD) and oppositional/defiant behavior [15] following cannabis exposure in the preconception, pre-or postnatal period (preconception, pre- or postnatal cannabis exposure [PCE]). While some studies point to a link between PCE and internalizing problems in children [13] others did not find any relation [14]. These psychiatric disorders are often preceded by early regulatory disorders.

Self-regulation is generally defined as goal-directed or -changing behavior to conform to external standards. Effortful self-regulation is often disaggregated into behavioral (e.g., executive functioning, attentional control) and emotional (e.g., emotion regulation) components [16]. However, regulatory disorders describe difficulties inappropriate to the age or developmental stage of the child in regulating own emotional states, independently or with the help of a caregiver. These difficulties must occur in one or more settings and last for at least one month [17]. Regulatory problems can present themselves in multiple different behaviors (e.g. sleeping, feeding or eating problems) [18, 19]. Clinically relevant regulatory disorders are assessed with the diagnostic systems DC: 0–5 or SIVA 0–6 and include, for example, dysregulated anger and aggression disorder or excessive crying [20, 21]. Further, they are characterized by reduced regulatory abilities, which are reflected in certain aspects of child temperament, such as regulatory functioning in infants [22] or behavioral activation and inhibition in young children [23]. As a result of early (multiple, persistent) regulation problems, an increased likelihood of developing internalizing and externalizing behavior problems has been observed [24]. Research suggests a cascade model in which early regulatory problems predict internalizing and externalizing problems in childhood [25], which in turn increase the risk of psychopathological symptoms in adolescence and adulthood [26].The relation between maternal substance use and child outcomes can be mediated in at least three different ways: (1) by noxious agents that cross the placental barrier and directly impact fetal brain development, (2) through neurohormonal changes induced by substance use, and (3) through maternal behavior and her relationship with the child. The placenta constitutes the immediate environment of the fetus, and therefore regulates the child’s exposure to environmental influences during pregnancy [27]. Research  indicates that all substances used by a woman during pregnancy pass through the placenta to some extent [28]. Besides direct exposure through the placenta, a child can be directly exposed to maternal substance use in the postnatal period through breastfeeding [5]. At the molecular level, epigenetic mechanisms such as DNA methylation and histone modification are assumed to play an important role in linking early adversities and child outcomes [29, 30]. Epigenetic alterations can affect, for example, immune function [31, 32], neurophysiological processes [33] as well as brain structure and function [34], which in turn could link prenatal substance exposure and child behavioral outcomes. Besides neurobiological mechanisms, changes in maternal behavior following early adversities can also affect the child’s behavior. Research suggests that maternal stress, psychopathology and also prenatal maternal substance use are related to impaired maternal parenting behaviors such as reduced responsivity or sensitivity in the mother–child-interaction [35–38]. Maternal parenting behavior in interaction with her child is associated with child self-regulation [39, 40]. Therefore, prenatal substance exposure may affect child behavioral outcomes through changes in maternal behavior in the mother–child-interaction.

To date, few studies have investigated neurobiological pathways mediating the relation between PCE and child behavioral and emotional problems. Findings from studies investigating effects of prenatal exposure to other substances indicate that neurobiological mechanisms play a crucial role in this association: PAE seems to elevate DNA methylation in stress-regulating genes and thereby increase the level of stress hormones [41] and changes in DNA methylation are assumed to mediate the relation between PAE and child outcomes [42]. Other studies suggest that DNA methylation is a mediating mechanism between PTE and child outcomes [43, 44]. An investigation in a small sample of children prenatally exposed to cocaine indicates that maternal crack cocaine intake might affect the methylation of child’s oxytocin receptors [45]. Changes in the hypothalamic–pituitary–adrenal (HPA) axis are discussed as another possible mechanism mediating the association between PAE and mental health problems in children [46, 47], and most studies reported elevated cortisol levels and a greater stress response in children after PAE [47, 48]. Brain changes such as structural differences have also been investigated in children prenatally exposed to substances. Alterations in fractional anisotropy (FA) and mean diffusivity were reported for children after PAE compared to unexposed controls [49–51]. Prenatal opioid exposure seems to be related to placental dysfunction and to affect fetal brain development [52], while prenatal methamphetamine exposure seems to be linked to structural brain changes, especially in striatal and hippocampal volume [53]. Prenatal substance exposure has been associated with child regulatory abilities and problems. For instance, PTE has been associated with lower motivational but not cognitive self-regulation [54] and with a decreased inhibitory control in preschoolers [55]. PAE has also been associated with self-regulatory problems in children [56], and children prenatally exposed to cocaine have been found to be associated with dysregulated emotions and behavior [57]. Although little is known about these mechanisms, prenatal substance exposure and child regulatory abilities and problems seem to be linked via neurobiological mechanisms: PTE has been found to be related with decreased placental NR3C1 methylation which in turn was associated with a decreased infant self-regulation and a greater need for handling to soothe the infant over the first month of life [58]. Alterations of child’s HPA axis functioning have been found to be related to impaired self-regulation [59], and PCE is also assumed to be related to sleep problems, hyperactivity and epigenetic changes [60].

To the best of our knowledge, no review has investigated the association between PCE and regulatory abilities and problems in young children. Previous systematic reviews have associated PCE with a broad range of child psychiatric disorders over childhood and adolescence [61], including externalizing problems [62], behavioral and cognitive outcomes in children [63], physical consequences for neonates [64] or neuropsychological outcomes [65] in children aged 6–18 years. Further, the underlying neurobiological mechanisms potentially mediating the associations between cannabis exposure and child regulatory abilities and problems remain unclear.

The goal of the present systematic review is to synthesize empirical research investigating associations between PCE and regulatory abilities and problems in children aged 0–6 years. Findings on these associations may contribute to a better understanding of the effects of maternal cannabis use and may incorporate into recommendations on cannabis use or cannabis abstinence for pregnant women, mothers and women in childbearing age in general. Temperamental characteristics are considered to be regulatory abilities, and regulatory disorders mentioned in DC: 0–5 and SIVA 0–6, are considered to be regulatory problems.

The age range of 0–6 years was chosen to cover the early childhood and in relation to the German diagnostic instrument SIVA 0–6 for the assessment of regulatory problems. Further, research suggests that regulatory problems often predispose later psychiatric disorders [66, 67]. Preventing and treating regulatory disorders in this age group therefore offers the opportunity to reduce the risk of psychiatric disorders across the lifespan, highlighting the important influence of this age group on later development. Additionally, we will include possible underlying neurobiological pathways that may help to explain the association. We have included neurobiological markers that have been shown to be altered in previous studies associated with maternal substance use. As studies report increased cannabis use not only during but also after pregnancy [8], the postnatal period should also be considered when investigating the effects of maternal cannabis use on the child. We will therefore include studies reporting effects of cannabis exposure in the preconception, pre-and postnatal period, as these are sensitive time windows for child development [34, 68, 69]. As cannabis and tobacco use are strongly correlated [70] and we assume that it would be difficult to include only studies on cannabis use, we do not exclude studies reporting on both cannabis and tobacco use.

## Method

**Fig. 1:**
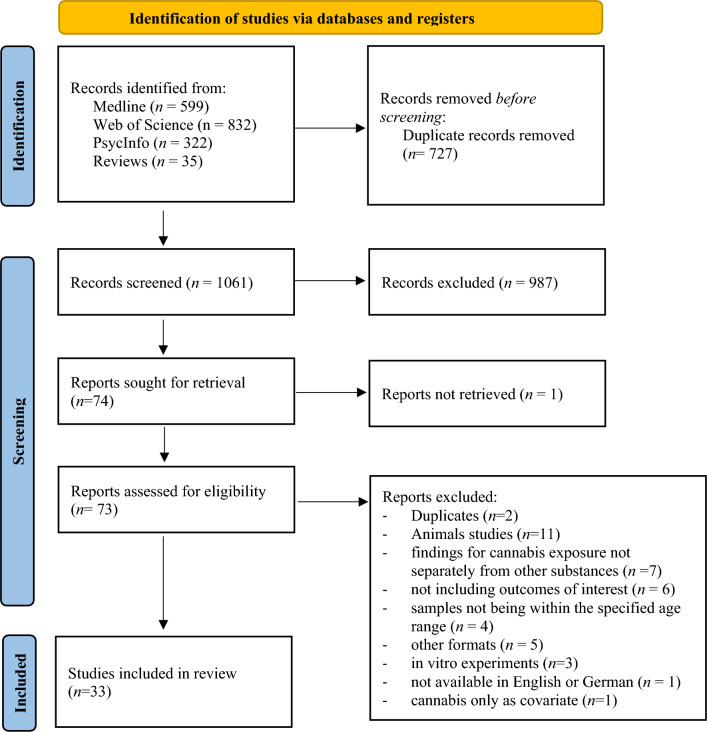
PRISMA flowchart of study selection process (adapted from Page et al. [71])

This systematic review was conducted and reported according to the PRISMA guidelines (see Fig. 1). It was previously registered in PROSPERO (ID: CRD42023425115).

### Search strategy

References were retrieved through electronic searches in Medline (Pubmed), Web of Science and PsycInfo from inception to 6 June 2023. Additionally, the reference lists of review articles were hand-searched for other potentially relevant references. For search terms and synonyms used in electronic searches see Table S1.

### Inclusion criteria

Studies had to meet the following criteria to be included in the present review:

1. Published in English or German in a peer-reviewed journal
2. Include a sample of children between 0 and 6 years of age with PCE
3. Investigate at least one outcome measure related to children’s regulatory abilities/problems or mediating neurobiological mechanisms
4. Report outcomes for children with no exposure to substances other than cannabis and tobacco in the preconception, pre-or postnatal period.

### Selection process

Two reviewers independently screened the titles and abstracts retrieved during the searches and identified all relevant studies. Disagreements were resolved through consensus or referral to a third reviewer where necessary. The agreement between the reviewers is reported. Relevant studies were then reviewed in full and reasons for exclusion were noted (see Table S2).

### Quality assessment

Two reviewers independently assessed the risk of bias in each included study using an adapted version of the Newcastle–Ottawa Scale, a standardized tool for rating the quality of cohort studies [72]. Disagreements over risk of bias in any study was resolved by discussion, or by consultation with a third team member, if required.

### Data collection process

Data extracted from the relevant publications include at a minimum (if available): author(s), year of publication, country, age of children, sample size, recruitment locations, maternal sociodemographic characteristics (age, race, education, relationship status), study design, time/duration of cannabis exposure, amount/frequency of cannabis exposure, method/material to assess cannabis exposure, relevant outcomes (regulatory abilities, regulatory problems, neurobiological mechanisms), control variables and findings.

## Results

Reviewer agreement on abstract screening was κ = 0.94 indicating an almost perfect interrater agreement [106]. After full text screening, *n* = 33 eligible studies were identified. Characteristics of included studies are shown in Tables 1, 2 and 3. Reasons for exclusion are reported in Fig. 1 and Table S2.

**Table 1:** Regulatory abilities

Study	Sample characteristics					Cannabis exposure			Regulatory abilities	Control variables	Findings
Authors (year), country	Age of children	Sample size	Recruitment	Maternal sociodemographic characteristics	Study design	Time/ duration	Amount/ frequency	Assessment method/material			
Stroud et al. [73] (2018), USA	0, 1, 2, 4, 5, 11, and 32 days	*n* = 24 PCE + PTE, *n* = 45 PTE, *n* = 42 unexposed	Obstetrical offices, health centers, and community postings during trimester 1 (t1) and t2	PCE + PTE: *M*_*age*_ = 25 years, 42% non-Hispanic white, 65% low socioeconomic status; PTE: *M*_*age*_ = 24 years, 53% non-Hispanic white, 47% low socioeconomic status; Unexposed: *M*_*age*_ = 25 years, 42% non-Hispanic white, 20% low socioeconomic status	Between-subject, longitudinal	Three months prior to conception and pregnancy	Average of 24 days over pregnancy; t1: 24 ± 24, t2: 1 ± 1, and t3: 0.04 ± 0.20 days	Maternal report; infant meconium	Self soothe (self-regulation), need for examiner soothing (handling), motor activity (lethargy) (NNNS)	Maternal demographics, medical conditions, depressive symptoms, alcohol and caffeine use; infant characteristics, tobacco exposure and feeding method	Decreased ability to self soothe (ß = − 0.357) and attend to stimuli (ß = − 0.626), increased need for examiner soothing (ß = .278) and low motor activity (ß = .136) in PCE + PTE compared to controls; effects on self-soothing (ß = − 0.185) and need for examiner soothing (ß = .112) stronger for PCE + PTE than PTE alone; stronger effects of PCE + PTE for females
De Moraes Barros et al. [74] (2006), Brazil	24–72 h	*n* = 26 PCE, *n* = 534 no PCE	Maternity hospital, adolescent mothers (10–20 years)	PCE: *M*_*age*_ = 16.5 years, 68% White, *M* = 6.8 years in school, 64% married No PCE: *M*_*age*_ = 16.9 years, 48% White, *M* = 7.3 years in school, 65% married	Between-subject, cross-sectional with prospective data collection	Pregnancy	n. a.	Interview, maternal hair, infant meconium	Arousal, regulation, handling, excitability, stress/abstinence signals (NNNS)	Demographic and birth data	Higher arousal (*r*^2^ = .061) and excitability (*r*^2^ = .0211) and lower regulation score (*r*^2^ = .007) in PCE compared to unexposed
Hoffmann et al. [75] (2020), USA	3 months	*n* = 98 no PCE,* n* = 26 PCE at conception, *n* = 13 PCE at conception, discontinued by GW 10,* n* = 25 PCE throughout pregnancy	Public safety-net prenatal clinic at 14–16 weeks gestation	No PCE: *M*_*age*_ = 30.9 years, 86% European, *M* = 14.2 years of education PCE at conception: *M*_*age*_ = 27.9 years, 73% European, *M* = 12.4 years of education PCE at conception, discontinued by GW 10: *M*_*age*_ = 26.9 years, 62% European, *M* = 11.9 years of education: PCE throughout pregnancy: *M*_*age*_ = 29.0 years, 84% European, *M* = 13.3 years of education	Between-subject, longitudinal	Conception and pregnancy	n. a.	Structured interviews, maternal urine	Temperament (IBQ-R)	Socio-economic, maternal health, and neonatal status parameters	Lower self-regulation after PCE throughout pregnancy (*d* = 0.79)
Ostlund et al. [76] (2021), USA	16 months	*n* = 69 no PTE/PCE,* n* = 81 PTE,* n* = 97 PCE + PTE	Local hospital at first prenatal appointment; smokers oversampled	No PTE/PCE *M*_*age*_ = 24.09 years, 51% African-American, *M* = 12.3 years of education, 465 married/living with partner	Between-subject, longitudinal	Pregnancy	n. a.	TLFB, maternal saliva, infant meconium	Temperament profile/self-regulatory abilities (TBAQ)	Maternal age, relationship status, education; infant sex, gestational age, birth weight, length	No direct association between PTE or PCE + PTE and infant temperament profile/self-regulatory abilities
Faden and Graubard [77] (2000), USA	3 years	*n* = 8 285	Live birth sample of the National and Maternal and Infant Health Survey (NMIHS) low-birthweight and black infants oversampled	n. a.	Between-subject, longitudinal	Pregnancy	n. a.	Self-report questionnaire	Level of happiness, activity level, difficult to manage (Denver Developmental scale, maternal report)	Mother and child demographics	No associations between PCE and outcomes
Hayes et al. [78] (1991), Jamaica	1, 3 and 30 days and 4–5 years	*n* = 30 PCE, *n* = 26 unexposed	Fieldwork	PCE: *M*_*age*_ = 22.9 years No PCE: *M*_*age*_ = 22.6 years	Between-subject, longitudinal	Pregnancy	n. a.	n. a.	Habituation, orientation, motor, range of state, regulation of state, autonomic stability, reflexes (NBAS)	n. a	Better autonomic regulation and reflexes in PCE group on day 30; no group differences in other ages
Eiden et al. [79] (2018), USA	24 months	*n* = 97 PCE + PTE, *n* = 81 PTE, *n* = 69 unexposed	Screening of all women in t1 presenting for prenatal care at a local hospital; tobacco users oversampled; racial diverse, mostly young, lower income and lower educational level	PCE: *M*_*age*_ = 23.8 years, 68% minority, *M* = 12.2 years of education No PCE: *M*_*age*_ = 24.5 years, 83% minority, *M* = 12.6 years of education	Between-subject, longitudinal	Pregnancy and postnatal (2, 9, 16 and 24 months)	Prenatal: *M* = 0.57 postnatal: *M* = 0.70 joints/day	Prenatal: TLFB, maternal oral fluid samples and infant meconium; postnatal: TLFB	Emotion regulation (5-min emotion regulation paradigm in laboratory)	n. a.	No direct association between PTE or PCE + PTE and emotion regulation
Murnan et al. [80] (2021), USA	3.5 years	*n* = 15 PCE, *n* = 48 no PCE	Delivery service for high- and low-risk obstetric patients	PCE: *M*_*age*_ = 26.8 years, 40% diploma/GED and 40% college, 93% not married No PCE: *M*_*age*_ = 28.1 years, 42% diploma/GED, 52% not married	Between-subject, longitudinal	Pregnancy	n. a.	Self-report, substance use information from obstetric medical record, maternal urine	Emotion regulation (Toy Behind Barrier task)	Child demographics, prenatal tobacco exposure; maternal/caregiver demographics and executive functioning	No group differences in emotion regulation
Eiden et al. [81] (2018), USA	2–3 years	*n* = 103 PCE + PTE, *n* = 75 PTE, *n* = 69 no PTE/PCE	Large city hospital during first prenatal appointment; tobacco smokers oversampled; young, unmarried, lower income, lower education, minority women	PCE: *M*_*age*_ = 23.5 years, 33% White, *M* = 12.2 years of education No PCE: *M*_*age*_ = 24.3 years, 22% White, *M* = 12.7 years of education	Between-subject, longitudinal	Pregnancy	t1: *M* = 0.65, t2: *M* = 0.21, t3: *M* = 0.21 joints/day	TLFB, maternal saliva, infant meconium	Emotional reactivity (CBCL 1.5–5, maternal report)	n. a.	No differences in emotional reactivity related to PCE/PTE
Moore et al. [15] (2023), USA	5 years	*n* = 6 PCE, *n* = 75 unexposed	Outpatient obstetric clinics prior to 24 GW	PCE: *M*_*age*_ = 26 years, 33% non-Hispanic white and 33% non-Hispanic black, 50% college or higher educational level No PCE: *M*_*age*_ = 31 years, 61% non-Hispanic white, 76% college or higher educational level	Between-subject, longitudinal	Pregnancy until 27 GW	n. a.	Maternal urine	Inhibitory control (Flanker test); emotional reactivity (CBCL)	Maternal demographics, height, weight and psychiatric illness	No group differences in inhibitory control and emotional reactivity
Parker et al. [82] (1990), USA	8–72 h	*n* = 259 PCE,* n* = 795 no PCE	Recruited after registration for prenatal care at City Hospital	66% Black, 41% not graduated from high school	Between-subject, longitudinal	Pregnancy	n. a.	Semi-structured interview, maternal urine	Jitteriness (NBAS)	n. a.	Positive association between PCE and neonatal jitteriness
Noland et al. [83] (2003), USA	4 years	*n* = 53 PCE, *n* = 116 unexposed in inhibition task	Large, urban, county-run hospital	n. a.	Between-subject, longitudinal	One month prior to pregnancy and pregnancy	n. a.	Maternal and fetal urine; self-report questionnaire	Inhibition (tapping inhibition task)	Maternal demographics; caregiver characteristics; number of prenatal visits; child birth date and IQ	No relationship between PCE and tap inhibition performance

*PCE* prenatal cannabis exposure, *PTE* prenatal tobacco exposure, *GW* gestational week, *t1* trimester 1, *t2* trimester 2, *t3* trimester 3, *NNNS* Neonatal Intensive Care Unit Network Neurobehavioral Scale, *NBAS* Brazelton Neonatal Behavioral Assessment Scales, *IBQ-R* Infant Behavior Questionnaire-Revised, *TBAQ* Toddler Behavior Assessment Questionnaire, *CBCL* Child Behavior Checklist, *TLFB* Timeline Follow-Back Interview, *n. a.* not available

**Table 2:** Studies on regulatory problems

Study	Sample characteristics					Cannabis exposure			Regulatory problems	Control variables	Findings
authors (year), country	Age of children	Sample size	Recruitment	Maternal sociodemographic characteristics	Study design	Time/duration	Amount/frequency	Assessment method/material			
Eiden et al. [81] (2018), USA	2–3 years	*n* = 103 PCE + PTE, *n* = 75 PTE, *n* = 69 no PTE/PCE	Large city hospital during first prenatal appointment; tobacco smokers oversampled; young, unmarried, lower income, lower education, minority women	PCE: *M*_*age*_ = 23.5 years, 33% White, *M* = 12.2 years of education No PCE: *M*_*age*_ = 24.3 years, 22% White, *M* = 12.7 years of education	Between-subject, longitudinal	Pregnancy	t1: *M* = 0.65, t2: *M* = 0.21, t3: *M* = 0.21 joints/day	TLFB, maternal saliva, infant meconium	Internalizing and externalizing problems (CBCL 1.5–5, maternal report)	n. a.	Fewer sleep problems in 2-year-old girls after PCE (r = − 0.22) No differences in aggressive behavior related to PCE/PTE for the whole sample
Murnan et al. [80] (2021), USA	3.5 years	*n* = 15 PCE, *n* = 48 no PCE	Delivery service for high- and low-risk obstetric patients	PCE: *M*_*age*_ = 26.8 years, 40% diploma/GED and 40% college, 93% not married No PCE: *M*_*age*_ = 28.1 years, 42% diploma/GED, 52% not married	Between-subject, longitudinal	Pregnancy	n. a.	Self-report, substance use information from obstetric medical record, maternal urine	Internalizing and externalizing problems (CBCL); aggressive behavior (Bobo Interaction Task)	Child demographics, prenatal tobacco exposure; maternal/caregiver demographics and executive functioning	No group differences in emotion regulation; CBCL scales: more aggressive behavior (ß = 5.73), sleep-related problems (ß = 2.08) and oppositional defiant behaviors (ß = 2.07) in PCE group; aggressive behavior task: among sub-group who engaged with doll, more aggressive behaviors in PCE group (ß = 0.74)
Dahl et al. [84] (1995), USA	3 years	*n* = 18 PCE, n = 20 controls (less than one joint per month)	Women from general obstetrical population: women with cannabis use of two or more joints per month and next women with lesser amount were selected	PCE: *M*_*age*_ = 23.3 years, *N* = 13 African-American, *M* = 11.6 years of education No PCE: *M*_*age*_ = 22.1 years, *N* = 9 African-American, *M* = 11.7 years of education	Between-subject, longitudinal	t1	Average amount during t1: 2.8 joints/day (range 0.3–5.0)	Interview	Sleep and arousal (total min of sleep, min in each sleep stage, min awake, number of arousals, latency to sleep onset, latency to first rapid eye movement period, and percentage of recording period spent asleep (sleep efficiency))	Demographic variables, alcohol, nicotine, or other substance exposure	No sign. differences sleep outcomes
Moore et al. [15] (2023), USA	5 years	*n* = 6 PCE, *n* = 75 unexposed	Outpatient obstetric clinics prior to 24 GW	PCE: *M*_*age*_ = 26 years, 33% non-Hispanic white and 33% non-Hispanic black, 50% college or higher educational level No PCE: *M*_*age*_ = 31 years, 61% non-Hispanic white, 76% college or higher educational level	Between-subject, longitudinal	Pregnancy until 27 GW	n. a.	Maternal urine	Internalizing and externalizing problems (CBCL, maternal report)	Maternal demographics, height, weight and psychiatric illness	Association between PCE and fewer internalizing problems; no group differences in sleep problems, aggressive behavior, oppositional/defiant
Faden and Graubard [77] (2000), USA	3 years	*n* = 8 285	Live birth sample of the National and Maternal and Infant Health Survey (NMIHS) Low-birthweight and black infants oversampled	n. a.	Between-subject, longitudinal	Pregnancy	Level of happiness: *M* = 0.07–0.10, activity level: *M* = 0.03–0.07, difficult to manage: 0.05–0.11 joints/ day	Self-report questionnaire	Eating problems, number of tantrums, (Denver Developmental scale, maternal report)	Mother and child demographics	No associations between PCE and outcomes
El Marroun et al. [85] (2011), Netherlands	18 months	*n* = 88 PCE, *n* = 435 PTE in early pregnancy, *n* = 276 PTE throughout pregnancy, *n* = 3278 no PTE/PCE	subsample of the Generation R Study Medium education, mainly Dutch origin	PCE: 63% Dutch origin; 51% secondary educational PTE in early pregnancy: 67% Dutch origin, 55% higher education PTE throughout pregnancy: 61% Dutch origin, 63% secondary education no PTE/PCE: 63% Dutch origin, 61% higher education	Between-subject, longitudinal	t1	n. a.	Self-report questionnaire	Anxiety/depression, attention problems, aggressive behavior scales (CBCL 1.5–5, maternal report)	Parental demographics, psychopathology; obstetric information	Association between PCE and increased scores on aggressive behavior scale in girls
Rompala, Nomura and Hurd [86] (2021), USA	3–6 years	*n* = 71 PCE, *n* = 251 unexposed	Sample from ongoing study, recruited from obstetrics clinics	PCE: *M*_*age*_ = 25.9 years, 27% college, 69% single No PCE: *M*_*age*_ = 28.5 years, 23% college, 42% single	Between-subject, longitudinal	Pregnancy	n. a.	Face-to-face evaluation	Aggression (Behavioral Assessment System for Children; BASC-2)	Parental demographics; maternal stress, anxiety, depression, and cigarette smoking; prenatal substance use, child’s sex and race	PCE associated with increased aggression; increased risk for clinically sign. problems with aggression (adjusted OR = 4.04); PCE × sex interaction: increased aggression in PCE group only in females
Godleski et al. [87] (2018), USA	24 and 36 months	*n* = 81 PTE, *n* = 97 PCE + PTE, *n* = 69 unexposed	first prenatal appointment in local area hospital; smoking oversampled	*M*_*age*_ = 24.1 years, 51% African-American, 30% less than high school education and 30% high school education, 45% not living with a partner	Between-subject, longitudinal	Pregnancy	PTE: *M* = 4.28 cigarettes/day; PCE + PTE: *M* = 5.56 cigarettes and *M* = 0.57 joints/day	TLFB, maternal oral fluid specimens, infant meconium	Externalizing problems (BITSEA)	Demographic risk; maternal aggressive disposition, warmth and sensitivity, affective dysregulation, current substance use; breastfeeding duration	No direct association between PTE or PCE + PTE and externalizing behavior

*PCE* prenatal cannabis exposure, *PTE* prenatal tobacco exposure, *GW* gestational week, *t1* trimester 1, *t2* trimester 2, *t3* trimester 3, *BITSEA* Brief Infant Toddler Social Emotional Assessment, *BASC-2* Behavioral Assessment System for Children, *CBCL* Child Behavior Checklist, *TLFB* Timeline Follow-Back Interview, *n. a.* not available

**Table 3:** Studies on neurobiological mechanisms

Authors (year), country	Sample characteristics					Cannabis exposure			Outcome measures	Control variables	Findings
	Age of children	Sample size	Recruitment	Maternal sociodemographic characteristics	Study design	Time/ duration	Amount/ frequency	Assessment method/material	Neurobiological mechanisms		
Fransquet et al. [88] (2017), Australia	8 weeks	*n* = 44 PCE,* n* = 760 unexposed	General public and specialist substance and alcohol antenatal services	PCE: *M*_*age*_ = 30.2 years, 82% Australian, 57% education year 12 or under, 66% living with partner No PCE: *M*_*age*_ = 32.6 years, 55% Australian, 16% education year 12 or under, 95% living with partner	Between-subject, longitudinal	Pregnancy, most common in t1	n. a.	Self-report questionnaires	DRD4 methylation	Other substance use, substance use at 8 weeks postpartum	PCE: very small increase in methylation at CpG.3 (adjusted for other substance use: β = 0.67) and increased methylation at CpG.21.22.23 (adjusted for PTE: β = 1.48; no sign. difference in methylation in PCE alone compared to PCE + PTE
Rompala, Nomura and Hurd [86] (2021), USA	3–6 years	*n* = 71 PCE, *n* = 251 unexposed	Mother–child dyads from ongoing study; recruited from obstetrics clinics at New York	PCE: *M*_*age*_ = 25.9 years, 27% college, 69% single No PCE: *M*_*age*_ = 28.5 years, 23% college, 42% single	Between-subject, longitudinal	Pregnancy	n. a.	Face-to-face evaluation	Steroid hormones (hair samples); transcriptome analysis of placental tissue	Parental demographics; maternal stress, state and trait anxiety, depression, and cigarette smoking; prenatal substance use, child’s sex and race	Increased cortisol levels in PCE children, no sign. group difference for cortisone; negative association between placental CB receptor 1 (CNR1) expression and weekly cannabis use; reduced placental expression of genes (type I interferon, neutrophil, and cytokine signaling pathways) involved in immune system function
DiNieri et al. [89] (2011), USA	18–22 weeks gestation	*n* = 24 PCE, *n* = 25 controls	Fetal brain specimens from saline-induced elective abortions	PCE: *M*_*age*_ = 22.3 years, *N* = 19 Black, *M* = 11.9 years of education No PCE: *M*_*age*_ = 23.8 years, *N* = 21 Black, *M* = 12.1 years of education	Between-subjects, cross-sectional	Pregnancy until GW 22	*M* = 1.24 ± 0.2 joints/day	Interview; Maternal urine and fetal meconium	Striatal dopamine and opioid-related genes (DRD2)	n. a.	Decreased DRD2 mRNA expression in NAc but not in putamen after PCE; negative correlation between NAc DRD2 mRNA levels and maternal report of cannabis use (*r* = − 0.42)
Wang et al. [90] (2004), USA	PCE: 20.14 ± 0.29 weeks, controls: 20.38 ± 0.22 weeks	Post-mortem fetal brain samples: *n* = 21 PCE, *n* = 21 unexposed	Women at midgestation term (GW 18–22) who planned voluntary saline-induced abortion	PCE: *M*_*age*_ = 23.2 years, *N* = 17 Black, *M* = 12.5 years of education Unexposed: *M*_*age*_ = 22.6 years, *N* = 17 Black, *M* = 13.2 years of education	Between-subjects, cross-sectional	Pregnancy until GW 22	n. a.. average joints/day: low: < .4 (n = 8); moderate: > .4 and < .89 (n = 4); heavy: > .89 (n = 6)	Interview; maternal urine and fetal meconium	Amygdala dopamine D2 gene expression	Fetal factors; fetal developmental measurements; substance exposure	Association between amount of PCE and reduction of D2 mRNA expression levels in amygdala basal nucleus (particularly in males) (*r* = − 0.461); no sign. PCE-related alterations in hippocampus or caudal striatum for D2, D1, and CB1 mRNA levels
Wang et al. [91] (2006), USA	PCE: 20.14 ± 0.29 weeks, controls: 20.38 ± .22	post-mortem fetal brain samples: *n* = 21 PCE,* n* = 21 unexposed	Women at midgestation term (GW 18–22) who planned voluntary saline-induced abortion	PCE: *M*_*age*_ = 23.2 years, *N* = 17 Black, *M* = 11.5 years of education Unexposed: *M*_*age*_ = 22.6 years, *N* = 17 Black, *M* = 12.2 years of education	Between-subjects, cross-sectional	Pregnancy until GW 22	Joints/day: 6 heavy (> .89), 4 moderate (> .4 and < .89), 8 light (< .4) users; 3 no self-reported but THC meconium positive	Interview; maternal urine and fetal meconium	Opioid-related genes in the fetal forebrain	Fetal factors; fetal developmental measurements; substance exposure	Association between PCE amount and increased μ receptor expression in amygdala (*r* = 0.40), and reduced preproenkephalin expression in caudal putamen (*p* = − 0.49); association between PCE and reduced κ receptor mRNA in mediodorsal thalamic nucleus; no association between PCE and mRNA expression of preprodynorphin, κand delta opioid receptor
Tortoriello et al. [92] (2014), USA	18–22 weeks gestation	*n* = 12 PCE, *n* = 12 controls	Fetal brain specimens from saline-induced elective abortions	n. a.	Between-subjects, cross-sectional	Pregnancy until GW 22	n. a.	Meconium	Cortical development, CB1 cannabinoid receptors (CB1)	Fetal age, body weight, foot length and cannabis exposure	Association between PCE and disruption of CB1 receptor signaling and reduced SCG10 expression in the cerebrum after PCE; association between SCG10 and axonal growth
Stroud et al. [93] (2020), USA	Seven assessments over first postnatal months: days 0, 1, 2, 4, 5, 11, and 32	*n* = 24 PCE + PTE, *n* = 45 PTE, *n* = 42 controls	Recruited from obstetrical offices, health centers, and community postings and enrolled during late t2 or t3; THC use only excluded	PCE + PTE: *M*_*age*_ = 25 years, 42% non-Hispanic white, 65% low socioeconomic status; PTE: *M*_*age*_ = 24 years, 53% non-Hispanic white, 47% low socioeconomic status; Unexposed: *M*_*age*_ = 25 years, 42% non-Hispanic white, 20% low socioeconomic status	Between-subject, longitudinal	Three months prior gestation and pregnancy	Days of use: *M* = 24 through pregnancy; t1: *M* = 24, t2: *M* = 0.5, and t3: *M* = 0.04	Adapted TLFB interview, meconium for THC	Saliva cortisol (baseline and stress response)	Maternal demographics, medical conditions, depressive symptoms, alcohol and caffeine use; infant characteristics, tobacco exposure and feeding method	BL: no sign. group effect; attenuated BL in PCE + PTE and PTE compared to controls (n.s.); attenuated BL levels in males in PCE + PTE compared to control and PTE (ß = − 0.436) Stress response: attenuated cortisol reactivity in PCE + PTE compared to controls (ß = − 0.250); attenuated cortisol reactivity for PCE + PTE compared to control males (ß = − 362); attenuated cortisol reactivity for PTE compared to control females (ß = − 0.351)
Eiden et al. [94] (2020), USA	Kindergarten age	*n* = 83 PCE + PTE, *n* = 67 PTE, *n* = 88 controls	Women at first prenatal care appointment at large urban prenatal clinic with smokers oversampled + 33 mother–child dyads recruited online at kindergarten age	PCE: *M*_*age*_ = 23.6 years, 69% minority, *M* = 12.2 years of education No PCE: *M*_*age*_ = 24.5 years, 83% minority, *M* = 12.6 years of education	Between-subject, longitudinal	Pregnancy and during childhood (2–36 months and after kindergarten began)	Prenatal: *M* = 0.61, postnatal: *M* = 0.69 joints/day	TLFB, maternal oral fluid samples, infant meconium	Infant cortisol reactivity (oral fluid samples before, during and after two frustration paradigms from school age version of Laboratory Temperament Assessment Battery (LABTAB) [95]	Demographic risk; birth outcomes; hours of sleep the night before saliva sample was collected; medication use; maternal age in t1	Overall lower levels of cortisol and sharp decrease in cortisol from pre- to post-stressor in PCE + PTE children (PCE + PTE as predictor of linear (β = − 0.07) and quadratic (β = 0.02) slope); slight increase before decline in controls
Josan et al. [96] (2022), USA	6–8 weeks	*n* = 22 PCE, *n* = 18 controls	Obstetrics clinics and birthing units; pregnant and recently postpartum women with PCE or without any substance use	PCE: *M*_*age*_ = 28.5 years, 55% college/university, 32% living with partner No PCE: *M*_*age*_ = 32 years, 56% college/university, 67% married	Between-subject, longitudinal	Pregnancy and between 6 and 8 weeks postnatal	n. a.	Self-report and milk samples	Levels of cannabinoids and SIgA in breast milk	Alcohol	Lower SIgA levels in milk of PCE compared to controls
Molnar et al. [97] (2018), USA	60 months	*n* = 17 PCE + PTE, *n* = 16 PTE, *n* = 12 controls	Recruited at first prenatal appointment in a local area hospital; smokers oversampled	PCE + PTE: *M*_*age*_ = 23.4 years, 29% Caucasian, *M* = 12.3 years of education PTE: *M*_*age*_ = 23.0 years, 57% Caucasian, *M* = 12.4 years of education Controls: *M*_*age*_ = 20.8 years, 25% Caucasian, *M* = 12.5 years of education	Between-subject, longitudinal	Pregnancy	Joints/day: t1: 0–5.07; t2: 0–2.54; t3: 0–2.77; 2 months postnatal: 0–0.91	TLFB; Maternal saliva specimens; Infant meconium and salivary cotinine	SIgA	n. a.	Higher SIgA levels in PCE + PTE (*d* = 1.35) and PTE (*d* = 0.93) compared to controls; PCE and PTE exposure or amount of exposure did not predict SIgA levels
Simon et al. [98] (2023), USA	Birth, and at 2, 9, 16, 24, 36, and 60 months	*n* = 68 PCE + PTE;* n* = 64 PTE; *n* = 79 controls	Recruited from a local hospital during first prenatal appointment; smokers oversampled	PCE + PTE: *M* = 12.24 years of education PTE: *M* = 12.62 years of education Controls: *M* = 12.73 years of education	Between-subject, longitudinal	Pregnancy and postnatally	Average joints/day (range): t1: 0.53 (0–6.88); t2: 0.11 (0–2.54); t3: 0.06 (0–2.77); postnatal: 0.22	TLFB; maternal salivary from each trimester; infant meconium	C-reactive protein (CRP) as an index of inflammation (child salivary samples) at 60 months	Child race, sex, body mass index, acute illness at 60-month assessment, history of breastfeeding, and maternal educational attainment	Maternal self-report: Interaction between PCE + PTE in t3 and child CRP concentrations (ß = 0.04); positive effect of PCE on CRP concentration at low PTE; main effect of PCE in t3 on CRP concentrations (ß = 0.55); no significant interactions or main effects of PCE and PTE in t1 and t2 and postnatally on CRP concentrations Infant meconium: lower CRP concentrations after late-term PCE + PTE compared to controls (ß = 0.27); positive association between postnatal cannabis exposure and CRP concentrations (ß = 0.15) Maternal self-report and biomarker: no differences in CRP concentrations across PCE + PTE, PTE and control groups; positive association between postnatal cannabis exposure and CRP concentrations (ß = 0.14); significant interaction between postnatal cannabis exposure and child sex (ß = 0.24): association between greater postnatal cannabis exposure and higher CRP concentrations in males
Bandoli et al. [99] (2021), USA	Delivery or during first year of life	*n* = 15,321 CRD, *n* = 3 037 957 no CRD, *n* = 6 705 CRD + nicotine, *n* = 7 086 CRD + Substance-related diagnosis	Population based cohort comprised of all births in California; analytical sample: live-born singletons between 2011–2017	CRD: *M*_*age*_ = 89% between 18–34 years, 38.1% Hispanics, 21% less than 12 years of education No CRD: *M*_*age*_ = 78% between 18–34 years, 49% Hispanics, 17% less than 12 years of education CRD + nicotine: *M*_*age*_ = 90% between 18–34 years, 46% Non-Hispanic White, 27% less than 12 years of education CRD + Substance-related diagnosis *M*_*age*_ = 87% between 18–34 years, 38% Non-Hispanic White, 32% less than 12 years of education	Retrospective	Pregnancy	n. a.	Health records made during pregnancy or delivery episode or birth record variables	Structural malformations in central nervous system	Maternal demographics, psychopathology, medical conditions and alcohol-related diagnosis	Associations between CRD (alone (RR = 1.2), with nicotine exposure (RR = 1.4) and with other substance-related diagnosis (RR = 1.6)) and central nervous system malformations
Peterson et al. [100] (2020), USA	37–46 weeks postmenstrual	*n* = 29 PCE, *n* = 29 cocaine, *n* = 18 methadone and/or heroin,* n* = 42 controls	Illicit substance–using pregnant women recruited from prenatal clinics and substance abuse treatment programs; Healthy pregnant women from prenatal clinics	PCE: *M*_*age*_ = 24.3 years, 48% Hispanic, *M* = 11.7 years of education Cocaine: *M*_*age*_ = 29.0 years, 42% Hispanic, *M* = 11.0 years of education; methadone and/or heroin *M*_*age*_ = 30.9 years, 50% Hispanic, *M* = 11.0 years of education; controls *M*_*age*_ = 25.9 years, 79% Hispanic, *M* = 12.5 years of education	Between-subject, longitudinal	Pregnancy	Average joints per trimester: t1: 205.1; t2: 99.4; t3: 27.1	Questionnaires and random urine toxicology screens during pregnancy and at delivery and medical record reviews	Anatomical imaging (MRI), DTI, T2 relaxometry, and magnetic resonance spectroscopic imaging	Newborn postmenstrual age at MRI, newborn sex, cumulative maternal tobacco and alcohol use during pregnancy, maternal demographics, depression severity, anxiety severity, or prenatal stress	Anatomic: dose-related volume reductions in several regions in PCE group DTI: association between PCE and alterations in fractional anisotropy (FA) and average diffusion coefficient in several regions Relaxometry and magnetic resonance spectroscopic imaging: associations between PCE and altered T2 relaxation times and N-acetylaspartate (NAA) concentration
Thomason et al. [101] (2021), USA	MRI between 22- and 39-weeks GA	*n* = 26 PCE, *n* = 42 controls	Recruited during routine obstetrical appointments in t2 and t3	PCE: *M*_*age*_ = 25.5 years, 81% African-American, 30.8% college, 53.8% single controls: *M*_*age*_ = 25.0 years, 83% African-American, 45.2% GED/high school diploma, 61.9% single	Between-subject, longitudinal	Pregnancy	n. a.	Urine toxicology; self-report	Hippocampal connectivity (MRI)	GA at scan	Association between PCE and weaker hippocampal connectivity to parietal, posterior cingulate cortex, anterior insula and left SFG and stronger hippocampal connectivity to frontocortical, particularly in dmPFC, right SFG and mPFC, left anterior temporal gyrus and motor cortex
Grewen, Salzwedel and Gao [102] (2015), USA	2–6 weeks	*n* = 20 PCE with or without alcohol, nicotine, SSRI, opiates, *n* = 23 exposure to combination of substances (no PCE), *n* = 20 controls	Subset of study from non-cocaine-exposed recruited in t3 from local obstetric clinics for low income women, local advertisements and Craigslist	n. a.	Between-subject, longitudinal	Pregnancy	Average joints per week: t1: 13.29; t2: 9.12; t3: 5.38; postnatal: 0.34	TLFB; perinatal medical record of prenatal urine toxicology and/or infant meconium	Brain connectivity (MRI)	GA at birth, postnatal age, birth weight, categorical substance exposure, socioeconomic status, maternal depressed affect	Hypo-connectivity in PCE group (right caudate–cerebellum; right caudate–occipital/fusiform; left caudate–cerebellum; left anterior insula–cerebellum) compared with no PCE and controls (no difference between no PCE and control group)
Salzwedel et al. [103] (2020), USA	2–6 weeks	*n* = 75 prenatal substance exposure (cocaine, *n* = 35 marijuana, alcohol, nicotine, SSRIs, and opioids), *n* = 58 unexposed	Women in t3 in residential and outpatient treatment programs for perinatal substance abuse; obstetric clinics, low-income obstetric clinic, flyers, advertisements, craigslist	n. a.	Between-subject, longitudinal	Pregnancy	n. a.	TLFB interview; questionnaire on PCE; medical record of prenatal urine toxicology	Brain connectivity (fMRI)	Sociodemographic data; scanner and motion parameters	Relationship between PCE and higher connectivity in medial/lateral parietal, sensorimotor, and orbital/lateral frontal regions
Scher et al. [104] (1988), USA	24–36h	*n* = 55 exposed, *n* = unexposed	Urban obstetrical hospital; selected if > 1 joints/day during t1	*M*_*age*_ = 22.2 years, 53% White, *M* = 11.8 years of education, 71% single	Between-subject, longitudinal	1 year before and pregnancy	Mean joints/day: t1: 0.78, t2: 0.38, t3: 0.32	Interview	EEG during sleep	Alcohol, marijuana, tobacco, other illicit substance use; maternal demographics; infant sex, birth weight, Dubowitz score, ponderal index; EEG technician	PCE in t1 predicted increased mixed active sleep (ß = 0.29), decreased low voltage irregular sleep (ß = − 0.33), decreased total quiet sleep (ß = − 0.41), decreased trace alternant (ß = − 0.46), increased small (ß = 0.31) and large (ß = 0.50) body movements PCE in t2 predicted decreased total quiet sleep (ß = − 0.43), decreased trace alternant (ß = − 0.32), increased large body movements (ß = 0.34) PCE in t3 predicted increased mixed active sleep (ß = 0.30), decreased total quiet sleep (ß = − 0.36), decreased trace alternant (ß = − 0.38), increased small (ß = 0.36) and large (ß = 0.57) body movements
Pollack et al. [105] (2021), USA	48 h	*n* = 30 PCE, *n* = 24 controls	PCE neonates in regional perinatal center, unexposed GA matched controls	PCE: *M*_*age*_ = 30 years Controls:* M*_*age*_ = 25 years	Between-subject, longitudinal	Pregnancy	n. a.	Self-report and/or maternal urine srug screening	Amplitude-integrated electroencephalogram (aEEG)	Maternal age, GA, THC level, tobacco use, infant and maternal urine drug screening, child demographics	PCE compared to control group had sign. aEEG abnormalities with absent sleep wake cycles; Umbilical cord substance levels (pg/g) were not correlated with abnormal aEEG
Dahl et al. [84] (1995), USA	3 years	*n* = 18 PCE, n = 20 controls (less than one joint per month)	Women from general obstetrical population: women with cannabis use of two or more joints per month and next women with lesser amount were selected;	PCE: *M*_*age*_ = 23.3 years, *N* = 13 African-American, *M* = 11.6 years of education No PCE: *M*_*age*_ = 22.1 years, *N* = 9 African-American, *M* = 11.7 years of education	Between-subject, longitudinal	t1	Average amount during t1: 2.8 joints/day (range 0.3–5.0)	Interview	EEG during sleep	Demographic variables, alcohol, nicotine, or other substance exposure	In PCE group lower sleep efficiency (Spearman’s ρ = − 0.41), more awake time and more frequent arousals after sleep onset (Spearman’s ρ = 0.46); no sign. differences in number of minutes in each sleep stage and latency to rapid eye movement period
Hoffman et al. [75] (2020), USA	3 months	*n* = 98 unexposed,* n* = 26 THC at conception, *n* = 13 PCE discontinued by GW10,* n* = 25 PCE over pregnancy	Public safety-net prenatal clinic at GW 14–16	no PCE: *M*_*age*_ = 30.9 years, 86% European, *M* = 14.2 years of education PCE at conception: *M*_*age*_ = 27.9 years, 73% European, *M* = 12.4 years of education PCE at conception, discontinued by GW 10: *M*_*age*_ = 26.9 years, 62% European, *M* = 11.9 years of education: PCE throughout pregnancy: *M*_*age*_ = 29.0 years, 84% European, *M* = 13.3 years of education	Between-subject, longitudinal	Conception and pregnancy	n. a.	Structured interviews Urine toxicology	Vertex electroencephalogram; maternal plasma choline and its metabolite betaine	Socio-economic, maternal health, and neonatal status parameters	Less inhibition with greater P50S2 amplitudes after PCE at GW10 or longer compared to unexposed (moderate effect) Decreased P50S2 amplitude through higher maternal choline levels No differences in infants exposed to THC during lactation

*PCE* prenatal cannabis exposure, *PTE* prenatal tobacco exposure, *THC* Tetrahydrocannabinol, *GW* gestational week, *GA* gestational age, *PD* postnatal day, *t1* trimester 1, *t2* trimester 2, *t3* trimester 3, *CRD* Cannabis-related diagnosis, *SC* synthetic cannabinoids, *CB* cannabinoid receptor, *SIgA* Secretory Immunoglobulin A, *CRP* C-reactive protein, *TLFB* Timeline Follow-Back Interview, *EEG* electroencephalography; *(f)MRI* (functional) magnetic resonance imaging, *DTI* diffusion tensor imaging, *SFG* superior frontal gyrus, *dmPFC* dorsomedial prefrontal cortex, *mPFC* medial prefrontal cortex, *SCG10* Superior Cervical Ganglion 10, *n. a.* not available

### Description of studies

All studies (*n* = 33) assessed prenatal maternal cannabis use, only *n* = 2 studies included maternal cannabis at conception and *n* = 5 studies additionally assessed cannabis exposure in the postnatal period. A total of *n* = 7 studies investigated effects of cannabis exposure on regulatory abilities, *n* = 2 studies on regulatory problems and *n* = 4 studies included measures on both regulatory abilities and problems. Neurobiological changes related to PCE were investigated in *n* = 17 of the included studies, *n* = 1 study assessed both neurobiological changes and regulatory abilities and *n* = 2 studies examined neurobiological changes as well as regulatory problems. Risk of bias is reported in Table S3. A total of 7 studies were assessed with an NOS score of 6, 11 studies each with a score of 5 and 4, 2 studies with a score of 3 and 1 study each with an NOS score of 2 and 1.

### Regulatory abilities

**Fig. 2:**
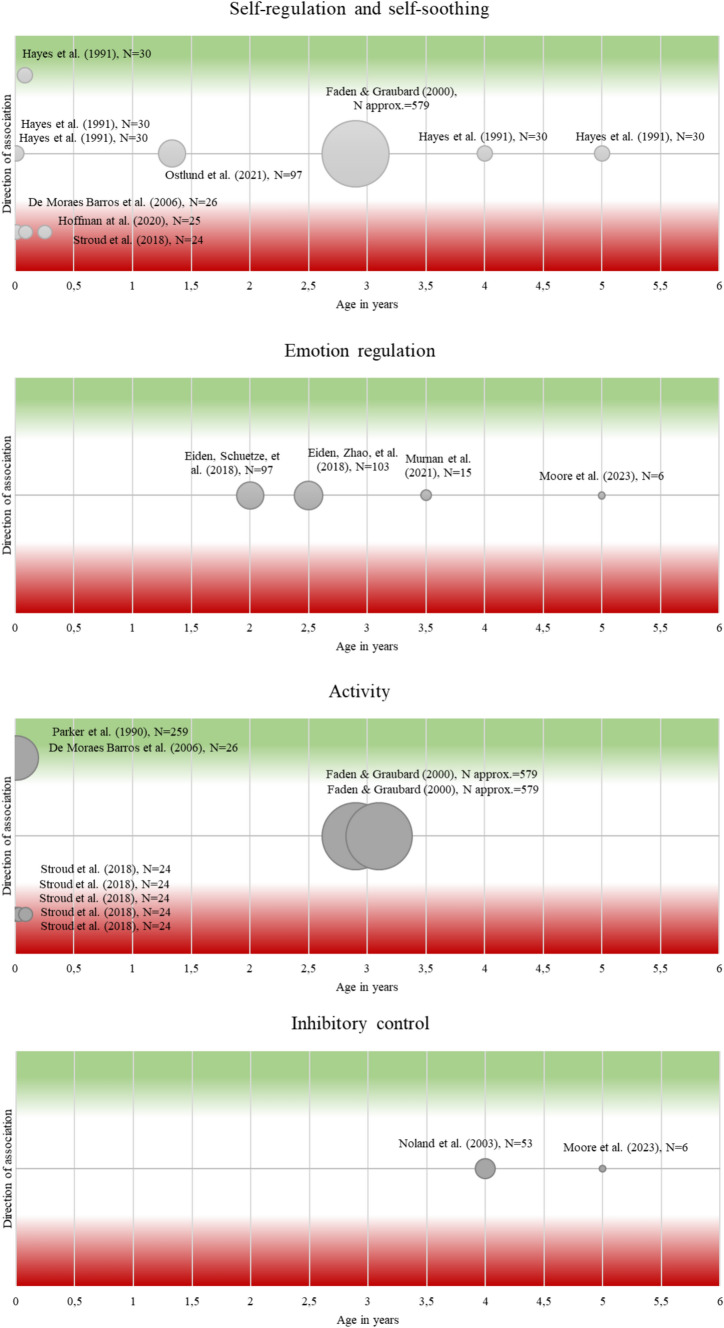
Comparison of studies with associations between PCE and regulatory abilities. *Notes:* X-axis = age of children during assessment. Y-axis = direction of association, with green indicating that PCE is associated with increased self-regulation and red indicating that PCE is associated with decreased self-regulation or dysregulation. Notably, only the direction of the association is presented, not the size of the association. Circle size is equivalent to the included number of children with PCE, with larger circles for studies with more PCE children. 1 = PCE associated with enhanced regulation, -1 = diminished regulation, 0 = no association. Faden and Graubard [77] did not report *N*_exposed_, therefore *N*_exposed_ was estimated based on US data on prenatal substance use [107], suggesting 7.0% of pregnant women reporting prenatal substance use, what results in approx. 8285*0.07 = 579 women with prenatal substance use

Table S4 and Fig. 2 present a comparison of studies regarding regulatory abilities and problems and their possible association with PCE.

#### Self-regulation and self-soothing

Stroud et al. [73] found a decreased ability to self-soothe and a higher need for external soothing in infants during their first month of life after cannabis exposure in the preconceptional or prenatal period. De Moraes Barros et al. [74] found lower regulatory ability in PCE neonates compared to non-exposed and Hoffman et al. [75] reported lower regulatory ability in 3-month-olds after PCE throughout pregnancy, but no difference regarding surgency and negativity. In contrast, results for older infants and toddlers showed that PCE was unrelated to self-regulatory abilities in 16-month-olds [76] and to soothability in 3-year-olds [77], 4-year-olds and 5-year-olds [78]. The latter study also found no associations between PCE and soothability, orientation and regulation in 1-, 3- and 30-day-olds [78].

#### Emotional regulation

Studies examining relations between PCE and emotion regulation found no association in 2-year-olds [79] and in 3.5-year-olds [80]. Also, no association was found between PCE and emotional reactivity in 3-year-olds [81] and in 5-year-olds [15].

#### Activity

For neonates, De Moraes Barros et al. [74] found increased arousal after PCE, and Parker et al. (1990) reported a positive association between PCE and jitteriness. Older infants up to one month of age have been reported to show less motor activity [73] after PCE + PTE than unexposed infants. In 3-year-olds, Faden and Graubard [77] found no association between PCE and activity in either direction.

#### Inhibitory control

Noland et al. [83] examined 4-year-olds and Moore et al. [15] 5-year-olds in a tap inhibition task. In both studies, no difference was found between PCE-exposed and non-exposed regarding inhibitory ability.

### Regulatory problems

**Fig. 3:**
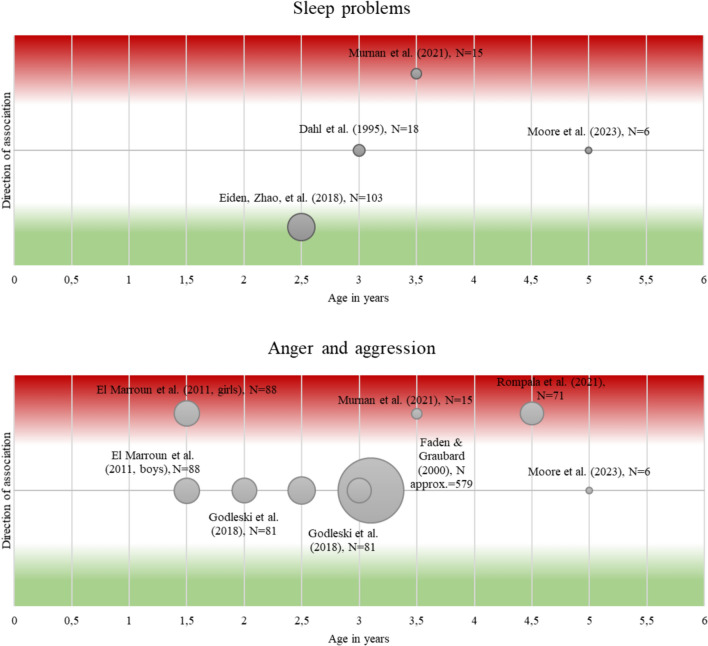
Comparison of studies with associations between PCE and several regulatory problems. *Notes:* Studies with excessive crying or eating problems as outcome are not presented given that either no or only one study was identified. X-axis = age of children during assessment. Y-axis = direction of association, with green indicating that PCE is associated with less regulatory problems and red indicating that PCE is associated with more regulatory problems. Notably, only the direction of the association is presented, not the size of the association. Circle size is equivalent to the included number of children with PCE, with larger circles for studies with more PCE children. 1 = PCE associated with more problems; − 1 = less problems; 0 = no association

Table S4 and Fig. 3 present a comparison of studies regarding regulatory problems and their possible association with PCE.

#### Sleep problems

In younger children, one study reported fewer sleep problems in 2-year-old girls after PCE [81], whereas another study found more sleep problems in 3.5-year-old girls after PCE [80]. No sleep problems were found in 3-year-olds after PCE compared with unexposed age-matched controls on various sleep variables such as total sleep time or sleep–wake schedules [84], and in 5-year-olds after PCE [15] compared with unexposed children.

#### Eating problems

The only identified study reported no association between PCE and eating problems in 3–year-olds [77].

#### Anger and aggression

Increased aggressive behavior but no differences in oppositional defiant behaviors (based on maternal reports) were found for 18-month-old girls after PCE [85]. Murnan et al. [80] observed more aggressive behavior of 3.5-year-olds in the PCE group and also described higher mother-reported aggressive and oppositional behavior. Rompala, Nomura and Hurd [86] investigated aggressive behavior in 3- to- 6-year-old children and reported increased aggression in children after PCE. No differences in maternal reports of aggressive child behavior and oppositional defiant child behavior were reported in 3-year-olds with versus without PCE [81] and in 5-year-olds [15]. Godleski et al. [87] reported no association between PTE or PCE + PTE and externalizing problems in 2- and 3-year-olds. Furthermore, no association was found between PCE and the number of tantrums in 3-year-olds [77].

### Neurobiological mechanisms

Five studies investigated *epigenetic* alterations related to PCE. Fransquet et al. [88] only found very small changes in DRD4 methylation in 8-week-old infants that did not survive correction for multiple testing. Rompala, Nomura and Hurd [86] examined placental tissue and found a negative correlation between weekly maternal prenatal cannabis use and CB1 expression of genes involved in immune system functioning. Four studies examined post-mortem fetal brain samples from abortions in gestation weeks 18–22: DiNieri et al. [89] found decreased DRD2 mRNA expression in the nucleus accumbens (NAc) but not in the putamen after PCE, as well as a negative correlation between NAc DRD2 mRNA levels and maternal report of cannabis use. Wang et al. [90] reported associations between PCE and D2 mRNA expression levels in the basal nucleus of the amygdala. Wang et al. [91] showed relations between PCE and increased μ receptor expression in the amygdala as well as reduced κ receptor mRNA levels in the mediodorsal thalamic nucleus, and reduced preproenkephalin expression in caudal putamen suggesting associations between PCE and opioid gene expression. Tortoriello et al. [92] described PCE-induced disruption of CB1 cannabinoid receptor expression which was related to increased phosphorylation of SCG10 through c-Jun N-terminal kinases. A reduction of SCG10 in turn was related to impaired axonal growth.

Three of the included studies assessed *cortisol* baseline level and reactivity in children after PCE (see Figure S1). Stroud et al. [93] examined infants at seven time points during their first postnatal month. Only male infants showed attenuated baseline cortisol levels after PCE + PTE compared to controls. Cortisol reactivity was attenuated in infants in the PCE + PTE group compared with controls, while there were no differences between PCE + PTE and PTE groups. Male infants showed an attenuated cortisol reactivity after PCE + PTE compared to controls, while no significant differences were found between PTE and control group. Females in the PTE group showed attenuated cortisol reactivity compared to controls, while there were no significant differences between females in PCE + PTE and control group. Rompala, Nomura and Hurd [86] investigated cortisol in hair samples of 3-to-6-year-olds and found increased cortisol levels in the PCE compared to the control group. Eiden et al. [94] examined kindergarten children and found overall lower cortisol levels and a sharp stress-induced increase in children in the PCE + PTE group while controls showed a slight increase.

Two studies investigated levels of *Secretory Immunoglobulin A* (SIgA) as an outcome of PCE effects. Josan et al. [96] reported lower SIgA levels in the breastmilk of mothers with cannabis intake during pregnancy compared to controls. Molnar et al. [97] investigated SIgA levels in saliva samples of 60-months-old children and found elevated SIgA levels in both PCE + PTE and PTE groups compared to controls. The study from Simon et al. [108] examined changes in salivary *C-reactive protein* related to PCE. Depending on the method used to assess PCE and PTE, results differed slightly. When PCE was assessed via maternal report, their findings revealed an interaction between PCE + PTE during the third trimester, but not during trimesters 1 and 2, and differences in CRP concentration at 60 months of age. Moreover, cannabis exposure was only positively associated with CRP concentrations at low tobacco exposure. Maternal reported postnatal cannabis and tobacco exposure, in contrast, were not related with CRP concentrations. The authors also analyzed associations between PCE + PTE assessed via infant meconium. Results from these analyses indicate lower CRP concentrations after late-term prenatal exposure to both cannabis and tobacco, while postnatal cannabis exposure was associated with higher CRP concentrations. Data from maternal report and biomarkers were also combined in this study to investigate differences between PCE + PTE, PTE and control group. Findings suggest that postnatal cannabis exposure is associated with higher CRP concentrations. Moreover, they reported a significant moderation through child sex indicating that greater postnatal cannabis exposure was related with higher CRP concentrations only in males.

Bandoli et al. [99] reported structural *brain malformations* in the central nervous system of infants after birth or during their first year of life associated with maternal cannabis-related disorders during pregnancy. Peterson et al. [100] found structural alterations in PCE infants compared to controls, demonstrating dose-related volume reductions in the dorsal and lateral surfaces of the frontal lobe, the mesial and inferior cerebral surfaces, and most of the lateral surface of the temporal lobe.

Studies examining *brain connectivity* in infants and children indicate alterations related to PCE, although the results are mixed. Thomason et al. [101] examined connectivity in fetuses between 22- and 39-weeks gestational age and reported PCE-associated weaker hippocampal connectivity to parietal lobe, posterior cingulate cortex, anterior insula, and right superior frontal gyrus and stronger connectivity between hippocampal and frontocortical regions, left anterior temporal gyrus and motor cortex. Peterson et al. [100] examined neonates 37–47 weeks postmenstrual and found associations between PCE and increased FA and reduced average diffusion coefficient in frontal and parietal white matter, increased FA in anterior limb of internal capsule and reduced FA in the posterior limb of the internal capsule. Further PCE was related to reduced T2 relaxation times in frontal and parietal white matter and with increased N-acetylaspartate (NAA) concentration in deep white matter of the frontal and parietal lobes. Grewen, Salzwedel and Gao [102] found hypo-connectivity between right caudate and both cerebellum and occipital/fusiform regions and between cerebellum and both left caudate and left anterior insula in 2-to-6-week-olds after PCE compared to both controls and infants exposed to other substances prenatally. Salzwedel et al. [103] also examined functional connectivity in 2-to-6-week old infants and found higher connectivity in medial/lateral parietal, sensorimotor, and orbital/lateral frontal regions related to PCE.

Several studies investigated functional PCE-related changes using *electroencephalography* (EEG). Scher et al. [104] reported relations between PCE and e.g. decreased quiet sleep and increased mixed active sleep in infants 24–36 h after birth and Pollack et al. [105] reported absence of sleep–wake-cycles in EEG in PCE infants 48 h after birth. Dahl et al. [84] examined 3-year-old infants using EEG during sleep and reported lower sleep efficiency, more awake time and more frequent arousals after sleep onset in PCE group compared to controls. No significant differences were found for duration of each sleep stage. Hoffman et al. [75] examined 1-month-old infants using vertex EEG and reported greater P50_S2_ amplitudes which indicate decreased inhibition in infants after PCE compared to unexposed. Cannabis exposure during lactation was not associated with alterations in inhibitory ability.

## Discussion

The aim of this systematic review was to synthesize empirical research on associations between PCE and regulatory abilities and problems in children aged 0–6 years, as regulatory problems at this age predict later psychiatric disorders [66, 67]. To gain insight into underlying mechanisms of action, we additionally considered possible neurobiological pathways.

Associations between PCE and *self-regulatory abilities* have been reported particularly for neonates and very young infants, i.e. regarding soothability and activity [73–75, 82]. Findings suggest that PCE seems to be especially related to self-regulation in infancy, whereas no associations were found from the age of about 2 years onwards. The limited ability to self-regulate as a result of PCE appears to be expressed primarily on the behavioral component of self-regulation. Nevertheless, it should be noted that only four studies could be included that investigated children's emotion regulation as a result of PCE and that no reliable conclusions can yet be drawn from the small number of studies. *Regulatory problems* may be associated with PCE in the areas of sleeping and aggressive behavior. In contrast to regulatory abilities, associations with regulatory problems, particularly sleep and aggression, appear to be more pronounced from around 2–3 years of age. It should be noted that findings on further problems, such as excessive crying, and their relations to PCE are lacking and require future investigations.

Altogether, findings suggest that there may be a stronger association between PCE and regulatory abilities in infants, while associations between PCE and regulatory problems appear to occur across the age range included. An explanation for this could be that deficits in regulatory abilities and problems manifest themselves differently as children grow older, for example in other psychopathological symptoms such as internalizing and externalizing problems. Since regulatory abilities and problems are considered to be precursors of later psychiatric disorders [66], a general dysregulation as a result of PCE could express itself in different psychopathology depending on developmental age. Recent studies provide evidence for such an association between regulatory abilities and problems in infants and toddlers and later emotional dysregulation in children [109, 110]. Dysregulation, as assessed with the CBCL dysregulation profile [111, 112], has been shown to be related to several behavioral problems in preschoolers [113] and dysregulation in childhood has been associated with psychopathology in adulthood [114]. Previous research suggests a close and likely bidirectional association between regulatory abilities and regulatory problems [115, 116], and both regulatory abilities and problems have been shown to be predictive of later internalizing and externalizing problems [25, 117] and therefore play a crucial role in psychopathology across lifespan [26]. Summing up, longitudinal studies with longer follow-ups are needed to investigate the developmental course of PCE-altered child self-regulation and the relation between regulatory abilities and problems in early childhood and both general dysregulation across the lifespan and its role in the development of later internalizing and externalizing problems. Another explanation for the fact that associations are found more frequently in infants and toddlers than in young children is the possible role of mother–child-interaction in the relation between PCE and child self-regulation. Self-regulation in the first years of life consists of co-regulation between child and caregiver. As children grow older, they take on an increasingly independent role in self-regulation [118]. Altered maternal behavior has been shown to be related to both PCE and child regulatory abilities and problems, and may be one of the variables that transmits the effects of PCE on child regulatory outcomes [37–40]. Ostlund et al. [76] reported an association between PCE + PTE and higher maternal hostility during pregnancy compared to controls. Moreover, hostility remained more stable in the PCE + PTE group than in the control group until 16 months after birth. Maternal hostility was again related to higher reactivity and dysregulation in the child. However, maternal hostility did not mediate the link between PCE + PTE and child self-regulation abilities. For future studies, it would be important to further disentangle the relation between PCE, maternal behavior and child regulatory outcomes, and to consider the role of maternal behavior as a potential mediator in this context. Maternal behavior as a potential mediator between PCE and child outcomes could play an important role in prevention and intervention programs to promote healthy development in exposed children and should therefore be considered in future research.

In this review, we investigated neurobiological pathways as mediating mechanism between PCE and children’s regulatory abilities and problems. Findings suggest PCE-related alterations in the opioid [91], endocannabinoid [92], and dopamine system as well as in dopamine receptor functioning [89, 90]. Although the results are mixed, the studies indicate gender differences and, consistent with other studies, report a higher responsiveness of substance-induced effects in males [90, 119]. The role of the dopamine system in the development of self-regulation has been investigated in previous studies. In particular, associations between different dopaminergic genotypes and a vulnerability for lower self-regulatory abilities and more problems have been reported [120]. Although epigenetic changes in the dopaminergic system were not investigated in these studies, they indicate an important role of changes in the dopaminergic system for the development of self-regulation and should therefore be considered in future studies. *Cortisol* reactivity appears to be increased in children prenatally exposed to PCE + PTE compared with controls [93, 94], while results on baseline cortisol levels were mixed [93, 94]. Overall, most results are consistent with previous studies reporting increased cortisol levels and reactivity following prenatal exposure to substances [47, 48] and are in line with assumptions of the HPA axis adaptation in response to prenatal adversities [121]. Clinical studies show that lower hair cortisol concentrations are associated with emotional symptoms in children aged 6–7 years, while behavioral problems are associated with higher hair cortisol concentrations in children aged 8–9 years [122]. Moreover, previous findings report associations between lower effortful control, as an aspect of regulatory ability, and stronger cortisol reactivity in 3-year-old children. They assumed that the stronger cortisol reactivity represents an enhanced HPA response resulting from poorer emotion regulation abilities [123]. However, it remains unclear whether the changes in cortisol levels are related to PCE or PTE or to the interaction of both substances. Previous studies on PTE-related alterations in cortisol levels in children also show mixed results [124, 125], therefore it would be important to investigate the effects of PTE, PCE and their interaction on the child’s stress system in future studies. Here, gender differences should also be considered. Only two studies assessed *SIgA* in children after PCE. The results suggest that children in the PCE group receive lower SIgA levels via breastmilk [96]. As the components of breastmilk have important effects on later health throughout life, changes in SIgA levels could have a longer-term impact on the development of the child's immune system [126]. However, previous studies have shown great heterogeneity in the composition of human breastmilk and therefore the results should be interpreted with caution. Molnar et al. [97] reported higher SIgA levels in both PCE + PTE and PTE groups compared to unexposed children aged 60 months. A recent review suggests that PCE may have adverse effects on the immune system of children, resulting in lower functioning [11]. In general, higher levels of SIgA are associated with chronic exposure to environmental toxins and recurrent infections [127]. To our best knowledge, few studies investigated associations between self-regulation and SIgA levels. Abraham, Zagoory-Sharon and Feldman [128] examined preschoolers and reported a negative association between self-regulatory abilities and SIgA levels. The findings of Simon et al. [108] also suggest an immunomodulatory effect of PCE. In particular, they indicate that PCE could only have a pro-inflammatory effect at low PTE levels. Furthermore, the results of this study imply that the timing of PCE may play a role in the inflammatory effects. In particular, cannabis exposure in the third trimester appears to have an effect on CRP concentrations in children. Previous studies suggest, for example, a relation between problems with self-regulation in childhood and higher CRP in adulthood [129] and a relation between CRP and emotion regulation in adolescence [130], but it remains unclear what role CRP might play in mediating the association between PCE and self-regulation. Taken together, the current findings suggest that PCE is associated with changes in immune functioning and that immune function may be a mediating factor of the relation between PCE and later self-regulation, but further research is needed to investigate these relations in more detail.

Two studies investigating alterations in *brain structure* were included. While Bandoli et al. [99] reported more general structural changes in children after PCE, Peterson et al. [100] looked more closely at structural alterations in specific brain regions. They demonstrated dose-related reductions and regional enlargement of several brain regions. These effects were very similar in children prenatally exposed to cocaine, and methadone and/or heroin, suggesting comparable effects on brain structure from prenatal exposure to different noxious substances. Volume reductions in brain regions have been shown, for example, for prenatal exposure to methamphetamine in neonates [131] and preschoolers [132]. However, overall findings are scarce and knowledge about the effects of prenatal substance exposure, particularly to cannabis, on brain structure alterations and associated behavioral outcomes in young children is still lacking.

Thomason et al. [101] reported altered *connectivity* in the fetal hippocampus in several brain regions during the third trimester, suggesting higher vulnerability for children prenatally exposed to cannabis and Salzwedel et al. [103] demonstrated partial evidence of associations between PCE-related functional brain alterations in neonates and problems with attention, memory, and executive control at 3 months of age. In particular, the studies reported here imply effects of PCE on connectivity in mPFC regions, motor cortex and insula [101–103]. Further, they suggest very similar effects of prenatal exposure to different substances on brain connectivity [100, 103]. Altogether, there is evidence that prenatal substance exposure affects functional connectivity in several brain regions. When considering brain regions involved in self-regulatory processes, several areas are assumed to be relevant, each associated with different facets of self-regulation [133, 134]. As the functional connectivity of the brain itself appears to be subject to developmental changes [134], exploring PCE-related changes and associations to behavioral outcomes is challenging, but could contribute to a better understanding of the underlying mechanisms of self-regulation and to develop early interventions. There are some limitations to this review that should be acknowledged. First, the quality between individual studies was variable. The quality of the studies assessing regulatory abilities is predominantly high (NOS score 5 or 6 out of 6). It appears that higher quality studies are more likely to indicate a (whether positive or negative) association with PCE, while all but one paper [75] of moderate quality (NOS score 3 or 4 out of 6) did not suggest a relation between PCE and regulatory abilities. In contrast, most studies assessing regulatory problems tend to be of moderate quality (mainly NOS score 4 out of 6), and most of them imply no relation between PCE and regulatory problems. This emphasizes the need for high-quality studies to obtain reliable results on associations between PCE and both regulatory abilities and problems. Studies investigating neurobiological mechanisms were predominantly of high quality (NOS scores 5 or 6 out of 6), with the exception of studies examining epigenetic changes and studies using EEG, which were predominantly of moderate study quality (mainly NOS score 4 out of 6). As the methods used and the outcomes assessed in the studies on neurobiological mechanisms vary widely, it is difficult to draw conclusions about links between results and study quality. However, it seems clear that more and higher quality studies should be conducted in the future to obtain reliable results and to understand the underlying mechanisms. Besides these differences in quality, the studies included in this review differed in their methodology, and the results should be interpreted with caution. In particular, we identified the following methodological issues: Many studies relied on maternal report to assess both PCE and child outcomes, which could lead to bias. As Simon et al. [98] reported, results seem to differ depending on the method used to assess maternal cannabis use. These findings underline the importance of using reliable methods in future studies. In addition, the use of different methods, such as questionnaires or observational paradigms, makes it difficult to compare results. Although the samples in most studies were representative, the studies with young children in particular consisted of a small number of participants, which weakens the validity of the results. In most studies, the extent of cannabis exposure was not assessed and/ or reported. In addition, the included studies did not assess and/or report the exact time of exposure, but only very broad time periods (e.g. pregnancy). Therefore, no conclusions can be drawn on the effects of the extent and timing of exposure on the relation between PCE and child self-regulation. As research on prenatal exposure to other substances such as alcohol and cocaine suggest that there may be dose-related effects [135, 136], future studies should take this aspect into account, especially when making recommendations for PCE. Probably the biggest limiting factor is that most of the studies did not capture confounding factors. In many studies, maternal self-regulation and psychopathology as well as exposure to substances other than cannabis and nicotine were not controlled. Only two of the studies included here assessed the cannabis use of the partners. Paternal cannabis use was not associated with child aggressive behaviour or attention problems [85]. Josan et al. [96] assessed the cannabis use of partners/ roommates of the pregnant women and did not find effects of their use on child immune function. In general, the consequences of secondhand or thirdhand smoke could also affect children postnatally, but these consequences have hardly been researched to date and should be addressed in future studies [137, 138]. It would also be interesting to investigate whether there are different consequences depending on the form in which a child is exposed to cannabis postnatally, i.e. via breast milk, via smoke through consumption by the parents or otherwise. Additionally, none of the studies included here assessed psychopathology of partners. Previous literature suggests, that paternal psychopathology, such as depression, during the pre- and postnatal period might have adverse effects for child emotional and behavioral development and should therefore be considered in future studies [139]. Other authors indicate that additional factors such as environmental characteristics, family life, income and education should be considerd as confounding variables [62, 140]. Studies also suggest that PCE is associated with later neurodevelopmental disorders, such as autism spectrum disorders and ADHD [141], the symptoms of which may in turn overlap with those of regulatory disorders in early childhood and should therefore be considered as potential confounding variables in future studies. This review emphasized the lack of research on associations between PCE and child self-regulation in general and studies on specific regulatory outcomes such as excessive crying in early childhood. Another limitation in assessing the effects of PCE is that cannabis use rarely occurs alone but usually in combination with tobacco smoking [70]. This review also includes some studies which only included children co-exposed to PCE + PTE and excluding those exposed to PCE only. Therefore, it often remains unclear which effects are associated with PCE, with PTE and with their interaction. 

To conclude, the findings reported here suggest adverse effects of PCE on child regulatory abilities and regulatory problems. Particularly, the findings indicate that there may be age-related differences and long-term investigations are needed to shed light on the question whether regulatory abilities and problems manifest themselves in other problems such as internalizing and externalizing symptoms, as children grow older, or whether there is a more general dysregulation as a result of PCE that underlies child psychopathology and expresses itself in different problem behaviors across different ages. Due to the insufficient number of studies, it was not possible to draw conclusions regarding the effects of cannabis exposure at different time points (preconception, pre- and postnatal) on outcomes in children and it would be interesting to consider this in future studies.

Our findings also suggest PCE-related neurobiological changes that may mediate the association between PCE and behavioral outcomes. There is evidence for gender-related differences in neurobiological changes and, in particular, PCE-related alterations of the HPA axis indicated by altered baseline cortisol levels and cortisol reactivity. Besides considering neurobiological pathways as potential mediating mechanisms in the association between PCE and child regulatory abilities and problems, there is evidence that maternal behavior and mother-child-interaction also play a crucial role. These potential underlying mechanisms should be investigated in more detail in future research.

Advanced knowledge on effects of PCE on child psychological development and underlying mechanisms would be important for making recommendations on cannabis use for pregnant women, women planning a pregnancy or even all women of childbearing age. Further, they form the basis for the development of prevention or intervention programs, as child regulatory abilities and problems cannot be addressed directly, but e.g. by promoting caregiver sensitivity and improving mother-child-interaction, which in turn may have a positive impact on child self-regulation [142]. Early interventions, especially in vulnerable mother-child dyads, e.g. after PCE, therefore offer the opportunity to improve the child's self-regulation and thus reduce the risk of later psychopathology and promote healthy psychosocial development in children.

## Supplementary Information

Below is the link to the electronic supplementary material.

Supplementary file1 (DOCX 312 KB)

## Data Availability

Not applicable.
