# Supplementary material for: Systematic review: the impact of maternal pre-and postnatal cannabis use on the behavioral and emotional regulation in early childhood
Source: Eur Child Adolesc Psychiatry. 2024 Jun 15;34(2):423–63. doi: 10.1007/s00787-024-02494-8 (PMC11868184; doi:10.1007/s00787-024-02494-8)
Supplement: Supplementary file 1 — Supplementary file1 (DOCX 312 KB) [file 787_2024_2494_MOESM1_ESM.docx]

**Title**: Systematic Review: The impact of maternal pre-and postnatal cannabis use on the behavioral and emotional regulation in early childhood

**Authors:** Emely Reyentanz^1*^, Dr. Jennifer Gerlach^2^, Dr. Sören Kuitunen-Paul^3,4,5^ and Prof. Dr. Yulia Golub^1^

**Affiliations:**^1^ Department of Child and Adolescent Psychiatry, Carl von Ossietzky Universität Oldenburg, Germany

^2^Department of Child and Adolescent Mental Health, University Hospital Erlangen, Friedrich-Alexander-Universität Erlangen-Nürnberg, Germany

^3^Chair of Clinical Child and Adolescent Psychology and Psychotherapy, Technische Universität Chemnitz, Chemnitz, Germany

^4^Chair of Clinical Psychology and Psychotherapy, Technische Universität Chemnitz, Chemnitz, Germany

^5^Chair of Child and Adolescent Psychiatry and Psychotherapy, Technische Universität Dresden, Dresden, Germany

***Corresponding Author**: Emely Reyentanz, emely.reyentanz(at)uol.de

**Table S1**

*Database Search Strategy*

| **Database** | Pubmed via Medline |
| --- | --- |
| **Search Query** | (newborn*[Title/Abstract]) OR (baby[Title/Abstract]) OR (babies[Title/Abstract]) OR (infant*[Title/Abstract]) OR (infancy[Title/Abstract]) OR (toddler*[Title/Abstract]) OR (toddlerhood[Title/Abstract]) OR (child*[Title/Abstract]) OR (children*[Title/Abstract]) OR (childhood[Title/Abstract]) OR (preschool*[Title/Abstract]) AND (Preconception*[Title/Abstract]) OR (Pregnant[Title/Abstract]) OR (pregnancy[Title/Abstract]) OR (prenatal*[Title/Abstract]) OR (perinatal*[Title/Abstract]) OR (postnatal*[Title/Abstract]) OR (maternal[Title/Abstract]) OR (mother*[Title/Abstract]) OR (in utero[Title/Abstract]) OR (placenta*[Title/Abstract]) AND (cannabis[Title/Abstract]) OR (cannabinoid*[Title/Abstract]) OR (THC[Title/Abstract]) OR (Delta-9 tetrahydrocannabinol[Title/Abstract]) OR (tetrahydrocannabinol[Title/Abstract]) OR (marijuana[Title/Abstract]) AND (regulation problem*[Title/Abstract]) OR (regulatory problem*[Title/Abstract]) OR (regulation disorder*[Title/Abstract]) OR (regulatory disorder*[Title/Abstract]) OR (regulation disturbance*[Title/Abstract]) OR (regulation difficulties[Title/Abstract]) OR (regulatory difficulties[Title/Abstract]) OR (regulation[Title/Abstract]) OR (regulated[Title/Abstract]) OR (dysregulation[Title/Abstract]) OR (dysregulatory[Title/Abstract]) OR (dysregulated[Title/Abstract]) OR (self regulation[Title/Abstract]) OR (self regulatory[Title/Abstract]) OR (self regulated[Title/Abstract]) OR (emotion regulation[Title/Abstract]) OR (emotion dysregulation[Title/Abstract]) OR (emotional regulation[Title/Abstract]) OR (emotional dysregulation[Title/Abstract]) OR (dysregulation profile[Title/Abstract]) OR (emotion*[Title/Abstract]) OR (effortful control[Title/Abstract]) OR (sleeping problem*[Title/Abstract]) OR (sleeping disturbance*[Title/Abstract]) OR (sleep*[Title/Abstract]) OR (feeding problem*[Title/Abstract]) OR (feed*[Title/Abstract]) OR (eating problem*[Title/Abstract]) OR (eat[Title/Abstract]) OR (crying problem*[Title/Abstract]) OR (anger problem*[Title/Abstract]) OR (anger dysregulation[Title/Abstract]) OR (anger[Title/Abstract]) OR (dysregulated anger[Title/Abstract] AND aggression disorder[Title/Abstract]) OR (adjustment disorder*[Title/Abstract]) OR (DC 0-3 diagnosis[Title/Abstract]) OR (DC 0-5 diagnosis[Title/Abstract]) OR (temperament*[Title/Abstract]) OR (mood[Title/Abstract]) OR (orient*[Title/Abstract]) OR (self-soothing[Title/Abstract]) OR (sooth*[Title/Abstract]) OR (inhibit*[Title/Abstract]) OR (hypersensitiv*[Title/Abstract]) OR (hyposensitiv*[Title/Abstract]) OR (activ*[Title/Abstract]) OR (hyperactiv*[Title/Abstract]) OR (reactiv*[Title/Abstract]) / newborn*[Title/Abstract]) OR (baby[Title/Abstract]) OR (babies[Title/Abstract]) OR (infant*[Title/Abstract]) OR (infancy[Title/Abstract]) OR (toddler*[Title/Abstract]) OR (toddlerhood[Title/Abstract]) OR (child*[Title/Abstract]) OR (children*[Title/Abstract]) OR (childhood[Title/Abstract]) OR (preschool*[Title/Abstract]) AND (Preconception*[Title/Abstract]) OR (Pregnant[Title/Abstract]) OR (pregnancy[Title/Abstract]) OR (prenatal*[Title/Abstract]) OR (perinatal*[Title/Abstract]) OR (postnatal*[Title/Abstract]) OR (maternal[Title/Abstract]) OR (mother*[Title/Abstract]) OR (in utero[Title/Abstract]) OR (placenta*[Title/Abstract]) AND (cannabis[Title/Abstract]) OR (cannabinoid*[Title/Abstract]) OR (THC[Title/Abstract]) OR (Delta-9 tetrahydrocannabinol[Title/Abstract]) OR (tetrahydrocannabinol[Title/Abstract]) OR (marijuana[Title/Abstract]) AND (Stress[Title/Abstract]) OR (Cortisol[Title/Abstract]) OR (HPA[Title/Abstract]) OR (stress reactivity[Title/Abstract]) OR (stress reaction[Title/Abstract]) OR (Glucocorticoid*[Title/Abstract]) OR (Hypothalamic-pituitary-adrenal[Title/Abstract]) OR (Hypothalamo-pituitary-adrenal[Title/Abstract]) OR (stress hormone[Title/Abstract]) / newborn*[Title/Abstract]) OR (baby[Title/Abstract]) OR (babies[Title/Abstract]) OR (infant*[Title/Abstract]) OR (infancy[Title/Abstract]) OR (toddler*[Title/Abstract]) OR (toddlerhood[Title/Abstract]) OR (child*[Title/Abstract]) OR (children*[Title/Abstract]) OR (childhood[Title/Abstract]) OR (preschool*[Title/Abstract]) AND (Preconception*[Title/Abstract]) OR (Pregnant[Title/Abstract]) OR (pregnancy[Title/Abstract]) OR (prenatal*[Title/Abstract]) OR (perinatal*[Title/Abstract]) OR (postnatal*[Title/Abstract]) OR (maternal[Title/Abstract]) OR (mother*[Title/Abstract]) OR (in utero[Title/Abstract]) OR (placenta*[Title/Abstract]) AND (cannabis[Title/Abstract]) OR (cannabinoid*[Title/Abstract]) OR (THC[Title/Abstract]) OR (Delta-9 tetrahydrocannabinol[Title/Abstract]) OR (tetrahydrocannabinol[Title/Abstract]) OR (marijuana[Title/Abstract]) AND (genome[Title/Abstract]) OR (gene*[Title/Abstract]) OR (SNP[Title/Abstract]) OR (Single nucleotide polymorphism[Title/Abstract]) OR (gene expression[Title/Abstract]) OR (mRNA[Title/Abstract]) OR (epigenetic*[Title/Abstract]) OR (genetic variant*[Title/Abstract]) OR (genetic variation*[Title/Abstract]) OR (DNA methylation[Title/Abstract]) OR (receptor[Title/Abstract]) OR (expression[Title/Abstract]) OR (receptor sensitivity[Title/Abstract]) OR (proteomics[Title/Abstract]) / newborn*[Title/Abstract]) OR (baby[Title/Abstract]) OR (babies[Title/Abstract]) OR (infant*[Title/Abstract]) OR (infancy[Title/Abstract]) OR (toddler*[Title/Abstract]) OR (toddlerhood[Title/Abstract]) OR (child*[Title/Abstract]) OR (children*[Title/Abstract]) OR (childhood[Title/Abstract]) OR (preschool*[Title/Abstract]) AND (Preconception*[Title/Abstract]) OR (Pregnant[Title/Abstract]) OR (pregnancy[Title/Abstract]) OR (prenatal*[Title/Abstract]) OR (perinatal*[Title/Abstract]) OR (postnatal*[Title/Abstract]) OR (maternal[Title/Abstract]) OR (mother*[Title/Abstract]) OR (in utero[Title/Abstract]) OR (placenta*[Title/Abstract]) AND (cannabis[Title/Abstract]) OR (cannabinoid*[Title/Abstract]) OR (THC[Title/Abstract]) OR (Delta-9 tetrahydrocannabinol[Title/Abstract]) OR (tetrahydrocannabinol[Title/Abstract]) OR (marijuana[Title/Abstract]) AND (brain[Title/Abstract]) OR (brain development[Title/Abstract]) OR (developmental changes[Title/Abstract]) OR (brain maturation[Title/Abstract]) OR (neogenesis[Title/Abstract]) OR (brain function*[Title/Abstract]) OR (MRI[Title/Abstract]) OR (fMRI[Title/Abstract]) OR (connectivity[Title/Abstract]) OR (diffusion tensor imaging[Title/Abstract]) OR (DTI[Title/Abstract]) |
| **Date Last Searched** | 06/06/2023 |
| **Articles Yielded** | 599 |

| **Database** | PsycINFO |
| --- | --- |
| **Search Query** | AB ( cannabis or cannabinoid* or THC or "Delta-9 tetrahydrocannabinol" or tetrahydrocannabinol or marijuana ) AND AB ( Preconception* or Pregnant or pregnancy or prenatal* or perinatal* or postnatal* or maternal or mother* or in utero or placenta* ) AND AB ( newborn* or baby or babies or infant* or infancy or toddler* or toddlerhood or child* or children* or childhood or preschool* )  AND AB ( "regulation problem*" or "regulatory problem*" or "regulation disorder*" or "regulatory disorder*" or "regulation disturbance*" or "regulation difficulties" or "regulatory difficulties" or regulation or regulated or dysregulation or dysregulatory or dysregulated or "self regulation" or "self regulatory" or "self regulated" or "emotion regulation" or "emotion dysregulation" or "emotional regulation" or "emotional dysregulation" or "dysregulation profile" or emotion* or "effortful control" or "sleeping problem*" or "sleeping disturbance*" or sleep* or "feeding problem*" or feed* or "eating problem*" or eat or "crying problem*" or "anger problem*" or "anger dysregulation" or anger or "dysregulated anger and aggression disorder" or "adjustment disorder*" or "DC 0-3 diagnosis" or "DC 0-5 diagnosis" or temperament* or mood or orient* or "self-soothing" or sooth* or inhibit* or hypersensitiv* or hyposensitiv* or activ* or hyperactiv* or reactiv* ) / AB ( cannabis or cannabinoid* or THC or "Delta-9 tetrahydrocannabinol" or tetrahydrocannabinol or marijuana ) AND AB ( Preconception* or Pregnant or pregnancy or prenatal* or perinatal* or postnatal* or maternal or mother* or in utero or placenta* ) AND AB ( newborn* or baby or babies or infant* or infancy or toddler* or toddlerhood or child* or children* or childhood or preschool* ) AND AB ( Stress or Cortisol or HPA or "stress reactivity" or "stress reaction" or Glucocorticoid* or "Hypothalamic-pituitary-adrenal" or "Hypothalamo-pituitary-adrenal" or "stress hormone*" ) / AB ( cannabis or cannabinoid* or THC or "Delta-9 tetrahydrocannabinol" or tetrahydrocannabinol or marijuana ) AND AB ( Preconception* or Pregnant or pregnancy or prenatal* or perinatal* or postnatal* or maternal or mother* or in utero or placenta* ) AND AB ( newborn* or baby or babies or infant* or infancy or toddler* or toddlerhood or child* or children* or childhood or preschool* ) AND AB ( genome or gene* or SNP or "Single nucleotide polymorphism" or "gene expression" or mRNA or epigenetic* or "genetic variant*" or "genetic variation*" or "DNA methylation” or receptor or expression or "receptor sensitivity" or proteomics ) / AB ( cannabis or cannabinoid* or THC or "Delta-9 tetrahydrocannabinol" or tetrahydrocannabinol or marijuana ) AND AB ( Preconception* or Pregnant or pregnancy or prenatal* or perinatal* or postnatal* or maternal or mother* or in utero or placenta* ) AND AB ( newborn* or baby or babies or infant* or infancy or toddler* or toddlerhood or child* or children* or childhood or preschool* ) AB ( brain or "brain development" or "developmental changes" or "brain maturation" or neogenesis or "brain function*" or MRI or fMRI or connectivity or "diffusion tensor imaging" or DTI ) |
| **Date Last Searched** | 06/06/2023 |
| **Articles Yielded** | 322 |

| **Database** | Web of Science |
| --- | --- |
| **Search Query** | TS=(Cannabis) OR TS=(cannabinoid*) OR TS=(THC) OR TS=(Delta-9 tetrahydrocannabinol) OR TS=(tetrahydrocannabinol) OR TS=(marijuana) AND (TS=(preconception*) OR TS=(pregnant) OR TS=(pregnancy) OR TS=(prenatal*) OR TS=(perinatal*) OR TS=(postnatal*) OR TS=(maternal) OR TS=(mother*) OR TS=(in utero) OR TS=(placenta*) AND TS=(newborn*) OR TS=(baby) OR TS=(babies) OR TS=(infant*) OR TS=(infancy) OR TS=(toddler*) OR TS=(toddlerhood) OR TS=(child*) OR TS=(children*) OR TS=(childhood) OR TS=(preschool*) AND TS=(regulation problem*) OR TS=(regulatory problem*) OR TS=(regulation disorder*) OR TS=(regulatory disorder*) OR TS=(regulation disturbance*) OR TS=(regulation difficulties) OR TS=(regulatory difficulties) OR TS=(regulation) OR TS=(regulated) OR TS=(dysregulation) OR TS=(dysregulatory) OR TS=(dysregulated) OR TS=(self regulation) OR TS=(self regulatory) OR TS=(self regulated) OR TS=(emotion regulation) OR TS=(emotion dysregulation) OR TS=(emotional regulation) OR TS=(emotional dysregulation) OR TS=(dysregulation profile) OR TS=(emotion*) OR TS=(effortful control) OR TS=(sleeping problem*) OR TS=(sleeping disturbance*) OR TS=(sleep*) OR TS=(feeding problem*) OR TS=(feed*) OR TS=(eating problem*) OR TS=(eat) OR TS=(crying problem*) OR TS=(anger problem*) OR TS=(anger dysregulation) OR TS=(anger) OR TS=(dysregulated anger and aggression disorder) OR TS=(adjustment disorder*) OR TS=(DC 0-3 diagnosis) OR TS=(DC 0-5 diagnosis) OR TS=(temperament*) OR TS=(mood) OR TS=(orient*) OR TS=(self-soothing) OR TS=(sooth*) OR TS=(inhibit*) OR TS=(hypersensitiv*) OR TS=(hyposensitiv*) OR TS=(activ*) OR TS=(hyperactiv*) OR TS=(reactiv*) / TS=(Cannabis) OR TS=(cannabinoid*) OR TS=(THC) OR TS=(Delta-9 tetrahydrocannabinol) OR TS=(tetrahydrocannabinol) OR TS=(marijuana) AND (TS=(preconception*) OR TS=(pregnant) OR TS=(pregnancy) OR TS=(prenatal*) OR TS=(perinatal*) OR TS=(postnatal*) OR TS=(maternal) OR TS=(mother*) OR TS=(in utero) OR TS=(placenta*) AND TS=(newborn*) OR TS=(baby) OR TS=(babies) OR TS=(infant*) OR TS=(infancy) OR TS=(toddler*) OR TS=(toddlerhood) OR TS=(child*) OR TS=(children*) OR TS=(childhood) OR TS=(preschool*) AND TS=(Stress) OR TS=(Cortisol) OR TS=(HPA) OR TS=(stress reactivity) OR TS=(stress reaction) OR TS=(Glucocorticoid*) OR TS=(Hypothalamic-pituitary-adrenal) OR TS=(Hypothalamo-pituitary-adrenal) OR TS=(stress hormone*) /  TS=(Cannabis) OR TS=(cannabinoid*) OR TS=(THC) OR TS=(Delta-9 tetrahydrocannabinol) OR TS=(tetrahydrocannabinol) OR TS=(marijuana) AND (TS=(preconception*) OR TS=(pregnant) OR TS=(pregnancy) OR TS=(prenatal*) OR TS=(perinatal*) OR TS=(postnatal*) OR TS=(maternal) OR TS=(mother*) OR TS=(in utero) OR TS=(placenta*) AND TS=(newborn*) OR TS=(baby) OR TS=(babies) OR TS=(infant*) OR TS=(infancy) OR TS=(toddler*) OR TS=(toddlerhood) OR TS=(child*) OR TS=(children*) OR TS=(childhood) OR TS=(preschool*) AND TS=(genome) OR TS=(gene*) OR TS=(SNP) OR TS=(Single nucleotide polymorphism) OR TS=(gene expression) OR TS=(mRNA) OR TS=(epigenetic*) OR TS=(genetic variant*) OR TS=(genetic variation*) OR TS=(DNA methylation) OR TS=(receptor) OR TS=(expression) OR TS=(receptor sensitivity) OR TS=(proteomics) / TS=(Cannabis) OR TS=(cannabinoid*) OR TS=(THC) OR TS=(Delta-9 tetrahydrocannabinol) OR TS=(tetrahydrocannabinol) OR TS=(marijuana) AND (TS=(preconception*) OR TS=(pregnant) OR TS=(pregnancy) OR TS=(prenatal*) OR TS=(perinatal*) OR TS=(postnatal*) OR TS=(maternal) OR TS=(mother*) OR TS=(in utero) OR TS=(placenta*) AND TS=(newborn*) OR TS=(baby) OR TS=(babies) OR TS=(infant*) OR TS=(infancy) OR TS=(toddler*) OR TS=(toddlerhood) OR TS=(child*) OR TS=(children*) OR TS=(childhood) OR TS=(preschool*) AND TS=(brain) OR TS=(brain development) OR TS=(developmental changes) OR TS=(brain maturation) OR TS=(neogenesis) OR TS=(brain function*) OR TS=(MRI) OR TS=(fMRI) OR TS=(connectivity) AND TS=(diffusion tensor imaging) OR TS=(DTI) |
| **Date Last Searched** | 06/06/2023 |
| **Articles Yielded** | 832 |

**Table S2**

*Excluded Articles and Reasons for Exclusion*

| **Article** | **Reasons for Exclusion** |
| --- | --- |
| 1. Almada, M., Alves, P., Fonseca, B., Carvalho, F., Queiros, C., Gaspar, H., Amaral, C., Teixeira, N., & Correia-da-Silva, G. (2020). Synthetic cannabinoids JWH-018, JWH-122, UR-144 and the phytocannabinoid THC activate apoptosis in placental cells. *TOXICOLOGY LETTERS*, *319*, 129-137. https://doi.org/10.1016/j.toxlet.2019.11.004 | In vitro experiment |
| 1. Beggiato, S., Ieraci, A., Tomasini, M., Schwarcz, R., & Ferraro, L. (2020). Prenatal THC exposure raises kynurenic acid levels in the prefrontal cortex of adult rats. *PROGRESS IN NEURO-PSYCHOPHARMACOLOGY & BIOLOGICAL PSYCHIATRY*, *100*. https://doi.org/10.1016/j.pnpbp.2020.109883 | Animal study |
| 1. Beiersdorf, J., Hevesi, Z., Calvigioni, D., Pyszkowski, J., Romanov, R., Szodorai, E., Lubec, G., Shirran, S., Botting, C., Kasper, S., Guy, G., Gray, R., Di Marzo, V., Harkany, T., & Keimpema, E. (2020). Adverse effects of Delta(9)-tetrahydrocannabinol on neuronal bioenergetics during postnatal development. *JCI INSIGHT*, *5*(23). <https://doi.org/10.1172/jci.insight.135418> | Animal study |
| 1. Bonnin, A., deMiguel, R., Castro, J., Ramos, J., & FernandezRuiz, J. (1996). Effects of perinatal exposure to Delta(9)-tetrahydrocannabinol on the fetal and early postnatal development of tyrosine hydroxylase-containing neurons in rat brain. *JOURNAL OF MOLECULAR NEUROSCIENCE*, *7*(4), 291-308. https://doi.org/10.1007/BF02737066 | Animal study |
| 1. Cajachagua-Torres, K., Jaddoe, V., de Rijke, Y., van den Akker, E., Reiss, I., van Rossum, E., & El Marroun, H. (2021). Parental cannabis and tobacco use during pregnancy and childhood hair cortisol concentrations. *DRUG AND ALCOHOL DEPENDENCE*, *225*. https://doi.org/10.1016/j.drugalcdep.2021.108751 | Did not meet age criteria |
| 1. Campolongo, P., Trezza, V., Cassano, T., Gaetani, S., Morgese, M. G., Ubaldi, M., Soverchia, L., Antonelli, T., Ferraro, L., Massi, M., Ciccocioppo, R., & Cuomo, V. (2007). Perinatal exposure to delta-9-tetrahydrocannabinol causes enduring cognitive deficits associated with alteration of cortical gene expression and neurotransmission in rats. *Addict Biol*, *12*(3-4), 485-495. <https://doi.org/10.1111/j.1369-1600.2007.00074.x> | Did not meet age criteria |
| 1. Conradt, E., Sheinkopf, S., Lester, B., Tronick, E., LaGasse, L., Shankaran, S., Bada, H., Bauer, C., Whitaker, T., Hammond, J., & Study, M. L. (2013). Prenatal Substance Exposure: Neurobiologic Organization at 1 Month. *JOURNAL OF PEDIATRICS*, *163*(4), 989-+. https://doi.org/10.1016/j.jpeds.2013.04.033 | findings not separately reported for cannabis |
| 1. Ellis, R., Bara, A., Vargas, C., Frick, A., Loh, E., Landry, J., Uzamere, T., Callens, J., Martin, Q., Rajarajan, P., Brennand, K., Ramakrishnan, A., Shen, L., Szutorisz, H., & Hurd, Y. (2022). Prenatal Delta (9)-Tetrahydrocannabinol Exposure in Males Leads to Motivational Disturbances Related to Striatal Epigenetic Dysregulation. *BIOLOGICAL PSYCHIATRY*, *92*(2), 127-138. https://doi.org/10.1016/j.biopsych.2021.09.017 | Did not meet age criteria |
| 1. Fried, P. (1995). The Ottawa Prenatal Prospective Study (OPPS) – Methodological issues and findings – its easy to throw the baby out with the bat water. *Life Sciences*, *56*(23-24), 2159-2168. <https://doi.org/10.1016/0024-3205(95)00203-I> | Summary of studies |
| 1. Fried, P. A., Watkinson, B., & Gray, R. (1992). A follow-up study of attentional behavior in 6-year-old children exposed prenatally to marijuana, cigarettes, and alcohol. *Neurotoxicology and Teratology*, *14*(5), 299-311. https://doi.org/10.1016/0892-0362(92)90036-A | No measures of outcomes of interest |
| 1. Griffith, D. R., Azuma, S. D., & Chasnoff, I. J. (1994). Three-year outcome of children exposed prenatally to drugs. *Journal of the American Academy of Child and Adolescent Psychiatry*, *33*(1), 20-27. https://doi.org/10.1097/00004583-199401000-00004 | not clearly stated how effects of marijuana were assessed in polydrug group |
| 1. Hanft, A., Burnham, M., Goodlin-Jones, B., & Anders, T. (2006). Sleep architecture in infants of substance-abusing mothers. *INFANT MENTAL HEALTH JOURNAL*, *27*(2), 141-151. https://doi.org/10.1002/imhj.20085 | findings not separately reported for cannabis |
| 1. Hansen, H., Krutz, B., Sifringer, M., Stefovska, V., Bittigau, P., Pragst, F., Marsicano, G., Lutz, B., & Ikonomidou, C. (2008). Cannabinolds enhance susceptibility of immature brain to ethanol neurotoxicity. *ANNALS OF NEUROLOGY*, *64*(1), 42-52. https://doi.org/10.1002/ana.21287 | Animal study |
| 1. Hayes, J. S., Lampart, R., Dreher, M. C., & Morgan, L. (1991). Five-year follow-up of rural Jamaican children whose mothers used marijuana during pregnancy. *The West Indian medical journal*, *40*(3), 120-123. | duplicate |
| 1. Herman, C., Clarke, T., & Drury, S. (2018). Prenatal marijuana exposure impacts infant emotional reactivity at 18 months in a polysubstance abuse model. *JOURNAL OF THE AMERICAN ACADEMY OF CHILD AND ADOLESCENT PSYCHIATRY*, *57*(10), S139-S139. https://doi.org/10.1016/j.jaac.2018.09.028 | Poster abstract |
| 1. Hinckley, J. D., & Dillon, J. (2022). Developmental impact. In P. Riggs & T. Thant (Eds.), *Cannabis in psychiatric practice: A practical guide.* (pp. 45-59). Springer Nature Switzerland AG. | Book |
| 1. Khoshnood-Shariaati, M., Ashrafzadeh, S., Dastjani-Farahani, A., Zamani, R., & Naseh, A. (2022). Comparison of Ocular and Brain Abnormalities Among Neonates With In Utero Exposure to Opium or Other Drugs. *Cureus*, *14*(8), e27648. https://doi.org/10.7759/cureus.27648 | findings not separately reported for cannabis |
| 1. Kumar, A. M., Solomon, J., Patel, V., Kream, R. M., Drieze, J. M., & Millard, W. J. (1986). Early exposure to delta 9-tetrahydrocannabinol influences neuroendocrine and reproductive functions in female rats. *Neuroendocrinology*, *44*(2), 260-264. https://doi.org/10.1159/000124654 | Animal study |
| 1. Leech, S. L., Richardson, G. A., Goldschmidt, L., & Day, N. L. (1999). Prenatal substance exposure: effects on attention and impulsivity of 6-year-olds. *Neurotoxicol Teratol*, *21*(2), 109-118. <https://doi.org/10.1016/s0892-0362(98)00042-7> | Did not meet age criteria |
| 1. Lester, B., Tronick, E., LaGasse, L., Seifer, R., Bauer, C., Shankaran, S., Bada, H., Wright, L., Smeriglio, V., Lu, J., Finnegan, L., & Maza, P. (2002). The maternal lifestyle study: Effects of substance exposure during pregnancy on neurodevelopmental outcome in 1-month-old infants. PEDIATRICS, 110(6), 1182-1192. https://doi.org/10.1542/peds.110.6.1182 | cannabis only as covariate |
| 1. Mahony, D. L., & Murphy, J. M. (1999). Neonatal drug exposure: assessing a specific population and services provided by visiting nurses. *Pediatric nursing*, *25*(1), 27-34, 108. | No full text |
| 1. Manduca, A., Servadio, M., Melancia, F., Schiavi, S., Manzoni, O., & Trezza, V. (2020). Sex-specific behavioural deficits induced at early life by prenatal exposure to the cannabinoid receptor agonist WIN55, 212-2 depend on mGlu5 receptor signalling. *BRITISH JOURNAL OF PHARMACOLOGY*, *177*(2), 449-463. https://doi.org/10.1111/bph.14879 | Animal study |
| 1. McLemore, G., & Richardson, K. (2016). Data from three prospective longitudinal human cohorts of prenatal marijuana exposure and offspring outcomes from the fetal period through young adulthood. *DATA IN BRIEF*, *9*, 753-757. https://doi.org/10.1016/j.dib.2016.10.005 | Summary of studies |
| 1. Mereu, G., Fa, M., Ferraro, L., Cagiano, R., Antonelli, T., Tattoli, M., Ghiglieri, V., Tanganelli, S., Gessa, G., & Cuomo, V. (2003). Prenatal exposure to a cannabinoid agonist produces memory deficits linked to dysfunction in hippocampal long-term potentiation and glutamate release. *PROCEEDINGS OF THE NATIONAL ACADEMY OF SCIENCES OF THE UNITED STATES OF AMERICA*, *100*(8), 4915-4920. <https://doi.org/10.1073/pnas.0537849100> | Animal study |
| 1. Miranda, C., Barata, T., Vaz, S., Ferreira, C., Quintas, A., & Bekman, E. (2020). hiPSC-Based Model of Prenatal Exposure to Cannabinoids: Effect on Neuronal Differentiation. *FRONTIERS IN MOLECULAR NEUROSCIENCE*, *13*. <https://doi.org/10.3389/fnmol.2020.00119> | In vitro experiment |
| 1. Mirochnick, M., Meyer, J., Frank, D., Cabral, H., Tronick, E., & Zuckerman, B. (1997). Elevated plasma norepinephrine after in utero exposure to cocaine and marijuana. *PEDIATRICS*, *99*(4), 555-559. https://doi.org/10.1542/peds.99.4.555 | findings not separately reported for cannabis |
| 1. Napiorkowski, B., Lester, B., Freier, M., Brunner, S., Dietz, L., Nadra, A., & Oh, W. (1996). Effects of in utero substance exposure on infant neurobehavior. *PEDIATRICS*, *98*(1), 71-75. | findings not separately reported for cannabis |
| 1. Nomura, Y., Ham, J., Pehme, P., Wong, W., Pritchett, L., Rabinowitz, S., Foldi, N., Hinton, V., Wickramaratne, P., & Hurd, Y. (2023). Association of maternal exposure to Superstorm Sandy and maternal cannabis use with development of psychopathology among offspring: the Stress in Pregnancy Study. *BJPSYCH OPEN*, *9*(3). https://doi.org/10.1192/bjo.2022.595 | findings not separately reported for cannabis and postnatal cannabis use until 24 months after births |
| 1. Notaras, M., Lodhi, A., Barrio-Alonso, E., Foord, C., Rodrick, T., Jones, D., Fang, H., Greening, D., & Colak, D. (2021). Neurodevelopmental signatures of narcotic and neuropsychiatric risk factors in 3D human-derived forebrain organoids. *MOLECULAR PSYCHIATRY*, *26*(12), 7760-7783. <https://doi.org/10.1038/s41380-021-01189-9> | In vitro experiment |
| 1. Pritchett, A. (2022). *Effects of prenatal cannabis exposure on offspring emotional development and stress response* ProQuest Information & Learning]. EBSCOhost. | Dissertation |
| 1. Rodríguez de Fonseca, F., Cebeira, M., Fernández-Ruiz, J. J., Navarro, M., & Ramos, J. A. (1991). Effects of pre- and perinatal exposure to hashish extracts on the ontogeny of brain dopaminergic neurons. *Neuroscience*, *43*(2-3), 713-723. https://doi.org/10.1016/0306-4522(91)90329-m | Animal study |
| 1. Saez, T., Aronne, M., Caltana, L., & Brusco, A. (2014). Prenatal exposure to teh CB1 and CB2 cannabinoid receptro agonist WIN 55,212-2 alters migration of early-born glutamatergic neurons and GABAergic interneurons in the rat cerebral cortex. *JOURNAL OF NEUROCHEMISTRY*, *129*(4), 637-648. https://doi.org/10.1111/jnc.12636 | Animal study |
| 1. Salzwedel, A., Grewen, K., Vachet, C., Gerig, G., Lin, W., & Gao, W. (2015). Prenatal Drug Exposure Affects Neonatal Brain Functional Connectivity. *JOURNAL OF NEUROSCIENCE*, *35*(14), 5860-5869. <https://doi.org/10.1523/JNEUROSCI.4333-14.2015> | duplicate |
| 1. Schuetze, P., Zhao, J., Eiden, R., Shisler, S., & Huestis, M. (2019). Prenatal exposure to tobacco and marijuana and child autonomic regulation and reactivity: An analysis of indirect pathways via maternal psychopathology and parenting. *DEVELOPMENTAL PSYCHOBIOLOGY*, *61*(7), 1022-1034. https://doi.org/10.1002/dev.21844 | No measures of outcomes of interest |
| 1. Shabani, M., Haghani, M., Sheibani, V., & Janahmadi, M. (2009). Changes in motor and learning behaviors of rats prenatally exposed to WIN 55212-2, a cannabinoid receptor agonist. *PHYSIOLOGY AND PHARMACOLOGY*, *13*(2), 120-129. | Not available in German or English |
| 1. Simon, S., Eiden, R., Molnar, D., Huestis, M., & Riis, J. (2023). Associations between prenatal and postnatal substance exposure and salivary C-reactive protein in early childhood. *NEUROTOXICOLOGY AND TERATOLOGY*, *95*. https://doi.org/10.1016/j.ntt.2022.107134 | No full text |
| 1. Smid, M., Metz, T., McMillin, G., Mele, L., Casey, B., Reddy, U., Wapner, R., Thorp, J., Saade, G., Tita, A., Miller, E., Rouse, D., Sibai, B., Costantine, M., Mercer, B., Caritis, S., & C, E. K. S. N. I. (2022). Prenatal Nicotine or Cannabis Exposure and Offspring Neurobehavioral Outcomes. *OBSTETRICS AND GYNECOLOGY*, *139*(1), 21-30. https://doi.org/10.1097/AOG.0000000000004632 | No measures of outcomes of interest |
| 1. Suárez, I., Bodega, G., Fernández-Ruiz, J., Ramos, J. A., Rubio, M., & Fernández, B. (2004). Down-regulation of the AMPA glutamate receptor subunits GluR1 and GluR2/3 in the rat cerebellum following pre- and perinatal delta9-tetrahydrocannabinol exposure. *Cerebellum*, *3*(2), 66-74. <https://doi.org/10.1080/14734220310017230> | Animal study |
| 1. Suárez, I., Bodega, G., Rubio, M., Fernández-Ruiz, J. J., Ramos, J. A., & Fernández, B. (2004). Prenatal cannabinoid exposure down- regulates glutamate transporter expressions (GLAST and EAAC1) in the rat cerebellum. *Dev Neurosci*, *26*(1), 45-53. <https://doi.org/10.1159/000080711> | Animal study |
| 1. Tchuente, V., Sheehy, O., Zhao, J., Gorgui, J., Gomez, Y., & Berard, A. (2022). Is in-utero exposure to cannabis associated with the risk of attention deficit with or without hyperactivity disorder? A cohort study within the Quebec Pregnancy Cohort. *BMJ OPEN*, *12*(8). https://doi.org/10.1136/bmjopen-2021-052220 | No measures of outcomes of interest |
| 1. Trezza, V., Campolongo, P., Cassano, T., Macheda, T., Dipasquale, P., Carratu, M., Gaetani, S., & Cuomo, V. (2008). Effects of perinatal exposure to delta-9-tetrahydrocannabinol on the emotional reactivity of the offspring: a longitudinal behavioral study in Wistar rats. *PSYCHOPHARMACOLOGY*, *198*(4), 529-537. https://doi.org/10.1007/s00213-008-1162-3 | No measures of outcomes of interest |
| 1. Wenger, T., Croix, D., Tramu, G., & Leonardelli, J. (1991). Effects of prenatally administered delta-9-tetrahydrocannabinol on hypothalamic neuropeptide-y content in rat offsprings. *Neuroendocrinology Letters*, *13*(1), 15-21. | No measures of outcomes of interest |

**Table S3**

*Study Quality Assessment using an adapted version of the Newcastle-Ottawa-Scale ^39^*

| **Study** | **Selection** | | | **Comparability** | **Outcome** | | **Score (of 6)** |
| --- | --- | --- | --- | --- | --- | --- | --- |
|  | Representativeness of the cases | Selection of the non-exposed cohort | Ascertainment of exposure | study controls for exposure to substances other than THC and nicotine | Assessment of outcome | Adequacy of follow up of cohorts |  |
| Bandoli et al.^40^ | * | * | * | * | * | NA | 5 |
| Dahl et al.^41^ | - | - | - | * | - | - | 1 |
| De Moraes Barros et al.^42^ | * | * | * | * | * | NA | 5 |
| DiNieri et al.^43^ | - | * | * | NR | * | NA | 4 |
| Eiden et al.^44^ | * | * | * | * | * | * | 6 |
| Eiden et al.^45^ | * | * | - | * | * | * | 5 |
| Eiden et al.^46^ | * | * | * | - | - | * | 4 |
| El Marroun et al.^47^ | * | * | - | * | - | * | 4 |
| Faden and Graubard^48^ | * | * | - | NR | - | * | 3 |
| Fransquet et al.^49^ | * | * | - | * | * | - | 4 |
| Godleski et al.^50^ | * | * | * | * | - | * | 5 |
| Grewen et al.^51^ | * | * | * | * | * | NR | 5 |
| Hayes et al.^15^ | * | * | - | * | * | * | 5 |
| Hoffman et al.^52^ | * | * | * | NR | - | * | 4 |
| Josan et al.^53^ | * | * | * | NR | * | * | 5 |
| Molnar et al.^54^ | * | * | * | * | * | * | 6 |
| Moore et al.^55^ | * | * | * | NR | - | * | 4 |
| Murnan et al.^56^ | * | * | * | NR | - | * | 4 |
| Noland et al.^57^ | * | * | * | * | * | * | 6 |
| Ostlund et al.^58^ | * | * | * | * | * | * | 6 |
| Parker et al.^59^ | * | * | * | NR | * | * | 5 |
| Peterson et al.^60^ | * | * | * | * | * | * | 6 |
| Pollack et al.^61^ | * | - | - | * | * | NA | 3 |
| Rompala et al.^62^ | * | * | - | * | - | * | 4 |
| Salzwedel et al.^63^ | * | * | * | * | * | NR | 5 |
| Scher et al.^64^ | * | * | - | * | * | NR | 4 |
| Simon et al.^65^ | * | * | * | * | * | NR | 5 |
| Stroud et al.^66^ | * | * | * | * | * | * | 6 |
| Stroud et al.^67^ | * | * | * | * | * | * | 6 |
| Thomason et al.^68^ | * | * | * | * | * | NA | 5 |
| Tortoriello et al.^69^ | - | NR | * | NR | * | NA | 2 |
| Wang et al.^70^ | - | * | * | * | * | NA | 4 |
| Wang et al.^71^ | - | * | * | * | * | NA | 4 |

*Note*. Each cell represents one question of the Newcastle-Ottawa-Scale ^39^. A star indicates that the criterion in question was fulfilled, while a hyphen indicates that the criterion was not fulfilled. NA = not applicable; NR = not reported. The scale was adapted for our purposes so that one selection criterion (“Demonstration that outcome of interest was not present at start of study”) and one outcome criterion (“Was follow-up long enough for outcomes to occur”) were not assessed, because studies often started during pregnancy when child self-regulation cannot yet be detected and because follow-up was not required as child self-regulation can be assessed immediately after birth.

**Table S4**

*Summary of main results on regulatory abilities and regulatory problems, including assessment of study quality*

|  | **Indicate a positive relation** | **Indicate no relation** | **Indicate a negative relation** |
| --- | --- | --- | --- |
| **Regulatory abilities** | | | |
|  | ***i.e., PCE is related to increased regulatory abilities*** |  | ***i.e., PCE is related to decreased regulatory abilities*** |
| Self-regulation and self-soothing | - Hayes et al.^15^ (30 days) | - Hayes et al.^15^ (1 and 3 days and 4 to 5 years) | - Stroud et al.^66^ (0, 1, 2, 4, 5, 11, and 32 days) |
|  |  | - Ostlund et al.^58^ (16 months) | - De Moraes Barros et al.^42^ (24 to 72 hours) |
|  |  | - Faden and Graubard^48^ (3 years) | - Hoffman et al.^52^ (3 months) |
| Emotion regulation |  | - Eiden et al.^44^ (24 months) |  |
|  |  | - Eiden et al.^46^ (3 years) |  |
|  |  | - Murnan et al.^56^ (3.5 years) |  |
|  |  | - Moore et al.^72^ (5 years) |  |
| Activity | - De Moraes Barros et al.^42^ (24 to 72 hours) | - Faden and Graubard^48^ (3 years) | - Stroud et al.^66^ (0, 1, 2, 4, 5, 11, and 32 days) |
|  | - Parker et al.^59^ (8 to 72 hours) |  |  |
| Inhibitory control |  | - Noland et al.^57^ (4 years) |  |
|  |  | - Moore et al.^72^ (5 years) |  |
| **Regulatory problems** | | | |
|  | ***i.e., PCE related to more regulatory problems*** |  | ***i.e., PCE related to less regulatory problems*** |
| Sleep | - Murnan et al.^56^ (3.5 years) | - Eiden et al.^46^ (boys; 2 to 3 years) | - Eiden et al.^46^ (girls; 2 to 3 years) |
|  |  | - Moore et al.^72,^Almeida et al.^73^(5 years) |  |
|  |  | - Dahl et al.^41^ (3 years) |  |
| Eating |  | - Faden and Graubard^48^ (3 years) |  |
| Anger and aggression | - El Marroun et al.^47^ (girls; 18 months) | - El Marroun et al.^47^ (boys; 18 months) |  |
|  | - Murnan et al.^56^ (3.5 years) | - Moore et al.^72^ (5 years) |  |
|  | - Rompala et al.^62^ (3-6 years) | - Faden and Graubard^48^ (3 years) |  |
|  |  | - Eiden et al.^46^ (3 years) |  |
|  |  | - Godleski et al.^50^ (24 and 36 months) |  |

| **Colour** | **NOS Score (of 6)** |
| --- | --- |
|  | 6 |
|  | 5 |
|  | 4 |
|  | 3 |
|  | 2 |
|  | 1 |

*Notes:*

**Figure S1**

*
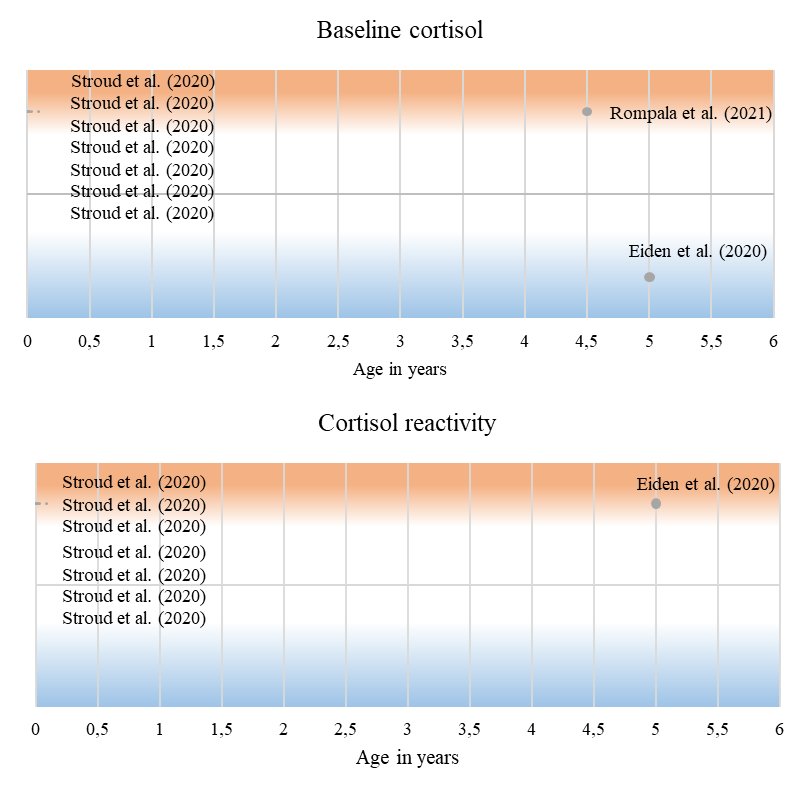
Comparison of studies with associations between PCE and baseline cortisol and cortisol reactivity*

*Notes:* X-axis = age of child ren during assessment. Y-axis = direction of association, with orange indicating a positive association and blue indicating a negative association between PCE and cortisol. Notably, only the direction of the association is presented, not the size of the association. Circle size is equivalent to the included number of children with PCE, with larger circles for studies with more PCE children. 1 = PCE associated with higher cortisol/stronger cortisol reactivity; -1 = lower cortisol/weaker cortisol reactivity; 0 = no association. Eiden et al.^45^ did not provide any more precise information on the age of the children than the ‘kindergarten age’, so an age of 5 years was assumed for the children in this study.

Literature

1. Almada M, Alves P, Fonseca B, et al. Synthetic cannabinoids JWH-018, JWH-122, UR-144 and the phytocannabinoid THC activate apoptosis in placental cells. *TOXICOLOGY LETTERS*. 2020 FEB 1 2020;319:129-137. doi:10.1016/j.toxlet.2019.11.004

2. Beggiato S, Ieraci A, Tomasini M, Schwarcz R, Ferraro L. Prenatal THC exposure raises kynurenic acid levels in the prefrontal cortex of adult rats. *PROGRESS IN NEURO-PSYCHOPHARMACOLOGY & BIOLOGICAL PSYCHIATRY*. 2020 JUN 8 2020;100doi:10.1016/j.pnpbp.2020.109883

3. Bonnin A, deMiguel R, Castro J, Ramos J, FernandezRuiz J. Effects of perinatal exposure to Delta(9)-tetrahydrocannabinol on the fetal and early postnatal development of tyrosine hydroxylase-containing neurons in rat brain. *JOURNAL OF MOLECULAR NEUROSCIENCE*. 1996 WIN 1996;7(4):291-308. doi:10.1007/BF02737066

4. Mereu G, Fa M, Ferraro L, et al. Prenatal exposure to a cannabinoid agonist produces memory deficits linked to dysfunction in hippocampal long-term potentiation and glutamate release. *PROCEEDINGS OF THE NATIONAL ACADEMY OF SCIENCES OF THE UNITED STATES OF AMERICA*. 2003 APR 15 2003;100(8):4915-4920. doi:10.1073/pnas.0537849100

5. Miranda C, Barata T, Vaz S, Ferreira C, Quintas A, Bekman E. hiPSC-Based Model of Prenatal Exposure to Cannabinoids: Effect on Neuronal Differentiation. *FRONTIERS IN MOLECULAR NEUROSCIENCE*. 2020 JUL 6 2020;13doi:10.3389/fnmol.2020.00119

6. Cajachagua-Torres K, Jaddoe V, de Rijke Y, et al. Parental cannabis and tobacco use during pregnancy and childhood hair cortisol concentrations. *DRUG AND ALCOHOL DEPENDENCE*. 2021 AUG 1 2021;225doi:10.1016/j.drugalcdep.2021.108751

7. Conradt E, Sheinkopf S, Lester B, et al. Prenatal Substance Exposure: Neurobiologic Organization at 1 Month. *JOURNAL OF PEDIATRICS*. 2013 OCT 2013;163(4):989-+. doi:10.1016/j.jpeds.2013.04.033

8. Ellis R, Bara A, Vargas C, et al. Prenatal Delta (9)-Tetrahydrocannabinol Exposure in Males Leads to Motivational Disturbances Related to Striatal Epigenetic Dysregulation. *BIOLOGICAL PSYCHIATRY*. 2022 JUL 15 2022;92(2):127-138. doi:10.1016/j.biopsych.2021.09.017

9. Fried PA, Watkinson B, Gray R. A follow-up study of attentional behavior in 6-year-old children exposed prenatally to marijuana, cigarettes, and alcohol. *Neurotoxicology and Teratology*. 1992-09 1992;14(5):299-311. doi:10.1016/0892-0362(92)90036-A

10. Griffith DR, Azuma SD, Chasnoff IJ. Three-year outcome of children exposed prenatally to drugs. *Journal of the American Academy of Child and Adolescent Psychiatry*. 1994 Jan 1994;33(1):20-27. doi:10.1097/00004583-199401000-00004

11. Hanft A, Burnham M, Goodlin-Jones B, Anders T. Sleep architecture in infants of substance-abusing mothers. *INFANT MENTAL HEALTH JOURNAL*. 2006 MAR 2006;27(2):141-151. doi:10.1002/imhj.20085

12. Hansen H, Krutz B, Sifringer M, et al. Cannabinolds enhance susceptibility of immature brain to ethanol neurotoxicity. *ANNALS OF NEUROLOGY*. 2008 JUL 2008;64(1):42-52. doi:10.1002/ana.21287

13. Herman C, Clarke T, Drury S. PRENATAL MARIJUANA EXPOSURE IMPACTS INFANT EMOTIONAL REACTIVITY AT 18 MONTHS IN A POLYSUBSTANCE ABUSE MODEL. *JOURNAL OF THE AMERICAN ACADEMY OF CHILD AND ADOLESCENT PSYCHIATRY*. 2018 OCT 2018;57(10):S139-S139. doi:10.1016/j.jaac.2018.09.028

14. Hinckley JD, Dillon J. Developmental impact. In: Riggs P, Thant T, eds. *Cannabis in psychiatric practice: A practical guide*. Springer Nature Switzerland AG; 2022:45-59. *Psychiatry update; ISSN: 2524-8316 (Print), 2524-8324 (Electronic)*.

15. Hayes JS, Lampart R, Dreher MC, Morgan L. Five-year follow-up of rural Jamaican children whose mothers used marijuana during pregnancy. *The West Indian medical journal*. 1991 1991;40(3):120-3.

16. Khoshnood-Shariaati M, Ashrafzadeh S, Dastjani-Farahani A, Zamani R, Naseh A. Comparison of Ocular and Brain Abnormalities Among Neonates With In Utero Exposure to Opium or Other Drugs. *Cureus*. 2022 Aug 2022;14(8):e27648. doi:10.7759/cureus.27648

17. Kumar AM, Solomon J, Patel V, Kream RM, Drieze JM, Millard WJ. Early exposure to delta 9-tetrahydrocannabinol influences neuroendocrine and reproductive functions in female rats. *Neuroendocrinology*. 1986 1986;44(2):260-264. doi:10.1159/000124654

18. Campolongo P, Trezza V, Cassano T, et al. Perinatal exposure to delta-9-tetrahydrocannabinol causes enduring cognitive deficits associated with alteration of cortical gene expression and neurotransmission in rats. *Addict Biol*. Sep 2007;12(3-4):485-95. doi:10.1111/j.1369-1600.2007.00074.x

19. Lester B, Tronick E, LaGasse L, et al. The maternal lifestyle study: Effects of substance exposure during pregnancy on neurodevelopmental outcome in 1-month-old infants. *PEDIATRICS*. 2002 DEC 2002;110(6):1182-1192. doi:10.1542/peds.110.6.1182

20. Mahony DL, Murphy JM. Neonatal drug exposure: assessing a specific population and services provided by visiting nurses. *Pediatric nursing*. 1999 Jan-Feb 1999;25(1):27-34, 108.

21. Manduca A, Servadio M, Melancia F, Schiavi S, Manzoni O, Trezza V. Sex-specific behavioural deficits induced at early life by prenatal exposure to the cannabinoid receptor agonist WIN55, 212-2 depend on mGlu5 receptor signalling. *BRITISH JOURNAL OF PHARMACOLOGY*. 2020 JAN 2020;177(2):449-463. doi:10.1111/bph.14879

22. McLemore G, Richardson K. Data from three prospective longitudinal human cohorts of prenatal marijuana exposure and offspring outcomes from the fetal period through young adulthood. *DATA IN BRIEF*. 2016 DEC 2016;9:753-757. doi:10.1016/j.dib.2016.10.005

23. Notaras M, Lodhi A, Barrio-Alonso E, et al. Neurodevelopmental signatures of narcotic and neuropsychiatric risk factors in 3D human-derived forebrain organoids. *MOLECULAR PSYCHIATRY*. 2021 DEC 2021;26(12):7760-7783. doi:10.1038/s41380-021-01189-9

24. Mirochnick M, Meyer J, Frank D, Cabral H, Tronick E, Zuckerman B. Elevated plasma norepinephrine after in utero exposure to cocaine and marijuana. *PEDIATRICS*. 1997 APR 1997;99(4):555-559. doi:10.1542/peds.99.4.555

25. Napiorkowski B, Lester B, Freier M, et al. Effects of in utero substance exposure on infant neurobehavior. *PEDIATRICS*. 1996 JUL 1996;98(1):71-75.

26. Nomura Y, Ham J, Pehme P, et al. Association of maternal exposure to Superstorm Sandy and maternal cannabis use with development of psychopathology among offspring: the Stress in Pregnancy Study. *BJPSYCH OPEN*. 2023 MAY 26 2023;9(3)doi:10.1192/bjo.2022.595

27. Pritchett A. *Effects of prenatal cannabis exposure on offspring emotional development and stress response*. ProQuest Information & Learning; 2022.

28. Rodríguez de Fonseca F, Cebeira M, Fernández-Ruiz JJ, Navarro M, Ramos JA. Effects of pre- and perinatal exposure to hashish extracts on the ontogeny of brain dopaminergic neurons. *Neuroscience*. 1991;43(2-3):713-23. doi:10.1016/0306-4522(91)90329-m

29. Saez T, Aronne M, Caltana L, Brusco A. Prenatal exposure to teh CB1 and CB2 cannabinoid receptro agonist WIN 55,212-2 alters migration of early-born glutamatergic neurons and GABAergic interneurons in the rat cerebral cortex. *JOURNAL OF NEUROCHEMISTRY*. 2014 MAY 2014;129(4):637-648. doi:10.1111/jnc.12636

30. Suárez I, Bodega G, Fernández-Ruiz J, Ramos JA, Rubio M, Fernández B. Down-regulation of the AMPA glutamate receptor subunits GluR1 and GluR2/3 in the rat cerebellum following pre- and perinatal delta9-tetrahydrocannabinol exposure. *Cerebellum*. 2004;3(2):66-74. doi:10.1080/14734220310017230

31. Suárez I, Bodega G, Rubio M, Fernández-Ruiz JJ, Ramos JA, Fernández B. Prenatal cannabinoid exposure down- regulates glutamate transporter expressions (GLAST and EAAC1) in the rat cerebellum. *Dev Neurosci*. Jan-Feb 2004;26(1):45-53. doi:10.1159/000080711

32. Schuetze P, Zhao J, Eiden R, Shisler S, Huestis M. Prenatal exposure to tobacco and marijuana and child autonomic regulation and reactivity: An analysis of indirect pathways via maternal psychopathology and parenting. *DEVELOPMENTAL PSYCHOBIOLOGY*. 2019 NOV 2019;61(7):1022-1034. doi:10.1002/dev.21844

33. Leech SL, Richardson GA, Goldschmidt L, Day NL. Prenatal substance exposure: effects on attention and impulsivity of 6-year-olds. *Neurotoxicol Teratol*. Mar-Apr 1999;21(2):109-18. doi:10.1016/s0892-0362(98)00042-7

34. Simon S, Eiden R, Molnar D, Huestis M, Riis J. Associations between prenatal and postnatal substance exposure and salivary C-reactive protein in early childhood. *NEUROTOXICOLOGY AND TERATOLOGY*. 2023 JAN 2023;95doi:10.1016/j.ntt.2022.107134

35. Smid M, Metz T, McMillin G, et al. Prenatal Nicotine or Cannabis Exposure and Offspring Neurobehavioral Outcomes. *OBSTETRICS AND GYNECOLOGY*. 2022 JAN 2022;139(1):21-30. doi:10.1097/AOG.0000000000004632

36. Tchuente V, Sheehy O, Zhao J, Gorgui J, Gomez Y, Berard A. Is in-utero exposure to cannabis associated with the risk of attention deficit with or without hyperactivity disorder? A cohort study within the Quebec Pregnancy Cohort. *BMJ OPEN*. 2022 AUG 2022;12(8)doi:10.1136/bmjopen-2021-052220

37. Trezza V, Campolongo P, Cassano T, et al. Effects of perinatal exposure to delta-9-tetrahydrocannabinol on the emotional reactivity of the offspring: a longitudinal behavioral study in Wistar rats. *PSYCHOPHARMACOLOGY*. 2008 JUL 2008;198(4):529-537. doi:10.1007/s00213-008-1162-3

38. Wenger T, Croix D, Tramu G, Leonardelli J. EFFECTS OF PRENATALLY ADMINISTERED DELTA-9-TETRAHYDROCANNABINOL ON HYPOTHALAMIC NEUROPEPTIDE-Y CONTENT IN RAT OFFSPRINGS. *NEUROENDOCRINOLOGY LETTERS*. 1991 FEB 1991;13(1):15-21.

39. Wells GA, Wells G, Shea B, et al. The Newcastle-Ottawa Scale (NOS) for Assessing the Quality of Nonrandomised Studies in Meta-Analyses. 2014:

40. Bandoli G, Jelliffe-Pawlowski L, Schumacher B, et al. Cannabis-related diagnosis in pregnancy and adverse maternal and infant outcomes. *DRUG AND ALCOHOL DEPENDENCE*. 2021 AUG 1 2021;225doi:10.1016/j.drugalcdep.2021.108757

41. Dahl RE, Scher MS, Williamson DE, Robles N, Day N. A longitudinal study of prenatal marijuana use. Effects on sleep and arousal at age 3 years. *Archives of pediatrics & adolescent medicine*. 1995 Feb 1995;149(2):145-150. doi:10.1001/archpedi.1995.02170140027004

42. De Moraes Barros M, Guinsburg R, Peres C, Mitsuhiro S, Chalem E, Laranjeira R. Exposure to marijuana during pregnancy alters neurobehavior in the early neonatal period. *JOURNAL OF PEDIATRICS*. 2006 DEC 2006;149(6):781-787. doi:10.1016/j.jpeds.2006.08.046

43. DiNieri JA, Wang X, Szutorisz H, et al. Maternal cannabis use alters ventral striatal dopamine D2 gene regulation in the offspring. *Biol Psychiatry*. Oct 15 2011;70(8):763-769. doi:10.1016/j.biopsych.2011.06.027

44. Eiden R, Schuetze P, Shisler S, Huestis M. Prenatal exposure to tobacco and cannabis: Effects on autonomic and emotion regulation. *NEUROTOXICOLOGY AND TERATOLOGY*. 2018 JUL 2018;68:47-56. doi:10.1016/j.ntt.2018.04.007

45. Eiden R, Shisler S, Granger D, Schuetze P, Colangelo J, Huestis M. Prenatal Tobacco and Cannabis Exposure: Associations with Cortisol Reactivity in Early School Age Children. *INTERNATIONAL JOURNAL OF BEHAVIORAL MEDICINE*. 2020 JUN 2020;27(3):343-356. doi:10.1007/s12529-020-09875-8

46. Eiden R, Zhao J, Casey M, Shisler S, Schuetze P, Colder C. Pre- and postnatal tobacco and cannabis exposure and child behavior problems: Bidirectional associations, joint effects, and sex differences. *DRUG AND ALCOHOL DEPENDENCE*. 2018 APR 1 2018;185:82-92. doi:10.1016/j.drugalcdep.2017.11.038

47. El Marroun H, Hudziak J, Tiemeier H, et al. Intrauterine cannabis exposure leads to more aggressive behavior and attention problems in 18-month-old girls. *DRUG AND ALCOHOL DEPENDENCE*. 2011 NOV 1 2011;118(2-3):470-474. doi:10.1016/j.drugalcdep.2011.03.004

48. Faden V, Graubard B. Maternal substance use during pregnancy and developmental outcome at age three. *JOURNAL OF SUBSTANCE ABUSE*. 2000 2000;12(4):329-340. doi:10.1016/S0899-3289(01)00052-9

49. Fransquet P, Hutchinson D, Olsson C, et al. Cannabis use by women during pregnancy does not influence infant DNA methylation of the dopamine receptor DRD4. *AMERICAN JOURNAL OF DRUG AND ALCOHOL ABUSE*. 2017 2017;43(6):671-677. doi:10.1080/00952990.2017.1314488

50. Godleski S, Shisler S, Eiden R, Huestis M. Co-use of tobacco and marijuana during pregnancy: Pathways to externalizing behavior problems in early childhood. *NEUROTOXICOLOGY AND TERATOLOGY*. 2018 SEP 2018;69:39-48. doi:10.1016/j.ntt.2018.07.003

51. Grewen K, Salzwedel A, Gao W. Functional connectivity disruption in neonates with prenatal marijuana exposure. *FRONTIERS IN HUMAN NEUROSCIENCE*. 2015 NOV 4 2015;9doi:10.3389/fnhum.2015.00601

52. Hoffman M, Hunter S, D'Alessandro A, Noonan K, Wyrwa A, Freedman R. Interaction of maternal choline levels and prenatal Marijuana's effects on the offspring. *PSYCHOLOGICAL MEDICINE*. 2020 JUL 2020;50(10):1716-1726. doi:10.1017/S003329171900179X

53. Josan C, Shiplo S, Fusch G, Raha S, Shea A. Cannabis use during lactation may alter the composition of human breast milk. *PEDIATRIC RESEARCH*. 2022 OCT 4 2022;doi:10.1038/s41390-022-02315-1

54. Molnar D, Granger D, Shisler S, Eiden R. Prenatal and postnatal cigarette and cannabis exposure: Effects on Secretory Immunoglobulin A in early childhood. *NEUROTOXICOLOGY AND TERATOLOGY*. 2018 MAY 2018;67:31-36. doi:10.1016/j.ntt.2018.03.003

55. Moore BF, Salmons KA, Hoyt AT, et al. Associations between Prenatal and Postnatal Exposure to Cannabis with Cognition and Behavior at Age 5 Years: The Healthy Start Study. *Int J Environ Res Public Health*. Mar 10 2023;20(6)doi:10.3390/ijerph20064880

56. Murnan A, Keim S, Yeates K, Boone K, Sheppard K, Klebanoff M. Behavioral and cognitive differences in early childhood related to prenatal marijuana exposure. *JOURNAL OF APPLIED DEVELOPMENTAL PSYCHOLOGY*. 2021 NOV 2021;77doi:10.1016/j.appdev.2021.101348

57. Noland J, Singer L, Arendt R, Minnes S, Short E, Bearer C. Executive functioning in preschool-age children prenatally exposed to alcohol, cocaine, and marijuana. *ALCOHOLISM-CLINICAL AND EXPERIMENTAL RESEARCH*. 2003 APR 2003;27(4):647-656. doi:10.1097/01.ALC.0000060525.10536.F6

58. Ostlund B, Perez-Edgar K, Shisler S, et al. Prenatal substance exposure and maternal hostility from pregnancy to toddlerhood: Associations with temperament profiles at 16 months of age. *DEVELOPMENT AND PSYCHOPATHOLOGY*. 2021 DEC 2021;33(5):1566-1583. doi:10.1017/S0954579421001000

59. Parker S, Zuckerman B, Bauchner H, Frank D, Vinci R, Cabral H. Jitteriness in full-term neonates: prevalence and correlates. *Pediatrics*. 1990 Jan 1990;85(1):17-23.

60. Peterson B, Rosen T, Dingman S, et al. Associations of Maternal Prenatal Drug Abuse With Measures of Newborn Brain Structure, Tissue Organization, and Metabolite Concentrations. *JAMA PEDIATRICS*. 2020 SEP 2020;174(9):831-842. doi:10.1001/jamapediatrics.2020.1622

61. Pollack R, Rana D, Purvis J, Pollard L, Pourcyrous M. Effect of prenatal marijuana exposure on sleep wake cycles and amplitude-integrated electroencephalogram (aEEG). *JOURNAL OF PERINATOLOGY*. 2021 JUN 2021;41(6):1355-1363. doi:10.1038/s41372-020-00911-9

62. Rompala G, Nomura Y, Hurd Y. Maternal cannabis use is associated with suppression of immune gene networks in placenta and increased anxiety phenotypes in offspring. *PROCEEDINGS OF THE NATIONAL ACADEMY OF SCIENCES OF THE UNITED STATES OF AMERICA*. 2021 NOV 23 2021;118(47)doi:10.1073/pnas.2106115118

63. Salzwedel A, Chen G, Chen Y, Grewen K, Gao W. Functional dissection of prenatal drug effects on baby brain and behavioral development. *HUMAN BRAIN MAPPING*. 2020 DEC 2020;41(17):4789-4803. doi:10.1002/hbm.25158

64. Scher MS, Richardson GA, Coble PA, Day NL, Stoffer DS. The effects of prenatal alcohol and marijuana exposure: disturbances in neonatal sleep cycling and arousal. *Pediatric research*. 1988 Jul 1988;24(1):101-105. doi:10.1203/00006450-198807000-00023

65. Simon SG, Eiden RD, Molnar DS, Huestis MA, Riis JL. Associations between prenatal and postnatal substance exposure and salivary C-reactive protein in early childhood. *Neurotoxicol Teratol*. Jan-Feb 2023;95:107-134. doi:10.1016/j.ntt.2022.107134

66. Stroud L, Papandonatos G, McCallum M, Kehoe T, Salisbury A, Huestis M. Prenatal tobacco and marijuana co-use: Impact on newborn neurobehavior. *NEUROTOXICOLOGY AND TERATOLOGY*. 2018 NOV 2018;70:28-39. doi:10.1016/j.ntt.2018.09.003

67. Stroud L, Papandonatos G, Jao N, Vergara-Lopez C, Huestis M, Salisbury A. Prenatal tobacco and marijuana co-use: Sex-specific influences on infant cortisol stress response. *NEUROTOXICOLOGY AND TERATOLOGY*. 2020 MAY 2020;79doi:10.1016/j.ntt.2020.106882

68. Thomason ME, Palopoli AC, Jariwala NN, et al. Miswiring the brain: Human prenatal Δ9-tetrahydrocannabinol use associated with altered fetal hippocampal brain network connectivity. *Developmental Cognitive Neuroscience*. 2021-10 2021;51doi:10.1016/j.dcn.2021.101000

69. Tortoriello G, Morris C, Alpar A, et al. Miswiring the brain: Delta(9)-tetrahydrocannabinol disrupts cortical development by inducing an SCG10/stathmin-2 degradation pathway. *EMBO JOURNAL*. 2014 APR 1 2014;33(7):668-685. doi:10.1002/embj.201386035

70. Wang X, Dow-Edwards D, Anderson V, Minkoff H, Hurd YL. In utero marijuana exposure associated with abnormal amygdala dopamine D2 gene expression in the human fetus. *Biol Psychiatry*. Dec 15 2004;56(12):909-15. doi:10.1016/j.biopsych.2004.10.015

71. Wang X, Dow-Edwards D, Anderson V, Minkoff H, Hurd YL. Discrete opioid gene expression impairment in the human fetal brain associated with maternal marijuana use. *Pharmacogenomics J*. Jul-Aug 2006;6(4):255-64. doi:10.1038/sj.tpj.6500375

72. Moore BF, Salmons KA, Hoyt AT, et al. Associations between Prenatal and Postnatal Exposure to Cannabis with Cognition and Behavior at Age 5 Years: The Healthy Start Study. *International journal of environmental research and public health*. 2023 Mar 10 2023;20(6)doi:10.3390/ijerph20064880

73. Almeida M, Dias-Rocha C, Reis-Gomes C, et al. Maternal high-fat diet impairs leptin signaling and up-regulates type-1 cannabinoid receptor with sex-specific epigenetic changes in the hypothalamus of newborn rats. *PSYCHONEUROENDOCRINOLOGY*. 2019 MAY 2019;103:306-315. doi:10.1016/j.psyneuen.2019.02.004
